# Supplementary material for: Vaccination Strategies Against Hepatic Diseases: A Scoping Review
Source: Vaccines (Basel). 2025 Dec 31;14(1):49. doi: 10.3390/vaccines14010049 (PMC12846253; doi:10.3390/vaccines14010049)
Supplement: Supplementary file 1 [file vaccines-14-00049-s001.zip › vaccines-4054386-supplementary.pdf]

Supplementary Material

Vaccination Strategies Against Hepatic Diseases: A Scoping Review

Zahra Beyzaei, Bita Geramizadeh, Sara Karimzadeh, Ralf Weiskirchen

Table S1. Prisma checklist

Scheme	ITEM	PRISMA-ScR CHECKLIST ITEM	REPORTED ON PAGE #
TITLE			
Title	1	Identify the report as a scoping review.	1
ABSTRACT			
Structured summary	2	Provide a structured summary that includes (as applicable): background, objectives, eligibility criteria, sources of evidence, charting methods, results, and conclusions that relate to the review questions and objectives.	1
INTRODUCTION			
Rationale	3	Describe the rationale for the review in the context of what is already known. Explain why the review questions/objectives lend themselves to a scoping review approach.	2
Objectives	4	Provide an explicit statement of the questions and objectives being addressed with reference to their key elements (e.g., population or participants, concepts, and context) or other relevant key elements used to conceptualize the review questions and/or objectives.	2
METHODS			
Protocol and registration	5	Indicate whether a review protocol exists; state if and where it can be accessed (e.g., a Web address); and if available, provide registration information, including the registration number.	3
Eligibility criteria	6	Specify characteristics of the sources of evidence used as eligibility criteria (e.g., years considered, language, and publication status), and provide a rationale.	3
Information sources*	7	Describe all information sources in the search (e.g., databases with dates of coverage and contact with authors to identify additional sources), as well as the date the most recent search was executed.	3
Search	8	Present the full electronic search strategy for at least 1 database, including any limits used, such that it could be repeated.	3
Selection of sources of evidence†	9	State the process for selecting sources of evidence (i.e., screening and eligibility) included in the scoping review.	3
Data charting process‡	10	Describe the methods of charting data from the included sources of evidence (e.g., calibrated forms or forms that have been tested by the team before their use, and whether data charting was done independently or in duplicate) and any processes for obtaining and confirming data from investigators.	3
Data items	11	List and define all variables for which data were sought and any assumptions and simplifications made.	3
Critical appraisal of individual sources of evidence§	12	If done, provide a rationale for conducting a critical appraisal of included sources of evidence; describe the methods used and how this information was used in any data synthesis (if appropriate).	3

Scheme	ITEM	PRISMA-ScR CHECKLIST ITEM	REPORTED ON PAGE #
Synthesis of results	13	Describe the methods of handling and summarizing the data that were charted.	3
**RESULTS**			
Selection of sources of evidence	14	Give numbers of sources of evidence screened, assessed for eligibility, and included in the review, with reasons for exclusions at each stage, ideally using a flow diagram.	3
Characteristics of sources of evidence	15	For each source of evidence, present characteristics for which data were charted and provide the citations.	3-15
Critical appraisal within sources of evidence	16	If done, present data on critical appraisal of included sources of evidence (see item 12).	3-15
Results of individual sources of evidence	17	For each included source of evidence, present the relevant data that were charted that relate to the review questions and objectives.	3-15
Synthesis of results	18	Summarize and/or present the charting results as they relate to the review questions and objectives.	3-15
**DISCUSSION**			
Summary of evidence	19	Summarize the main results (including an overview of concepts, themes, and types of evidence available), link to the review questions and objectives, and consider the relevance to key groups.	15-21
Limitations	20	Discuss the limitations of the scoping review process.	21
Conclusions	21	Provide a general interpretation of the results with respect to the review questions and objectives, as well as potential implications and/or next steps.	21
**FUNDING**			
Funding	22	Describe sources of funding for the included sources of evidence, as well as sources of funding for the scoping review. Describe the role of the funders of the scoping review.	22

JBI = Joanna Briggs Institute; PRISMA-ScR = Preferred Reporting Items for Systematic reviews and Meta-Analyses extension for Scoping Reviews.

* Where sources of evidence (see second footnote) are compiled from, such as bibliographic databases, social media platforms, and Web sites.

† A more inclusive/heterogeneous term used to account for the different types of evidence or data sources (e.g., quantitative and/or qualitative research, expert opinion, and policy documents) that may be eligible in a scoping review as opposed to only studies. This is not to be confused with information sources (see first footnote).

‡ The frameworks by Arksey and O'Malley (6) and Levac and colleagues (7) and the JBI guidance (4, 5) refer to the process of data extraction in a scoping review as data charting.

§ The process of systematically examining research evidence to assess its validity, results, and relevance before using it to inform a decision. This term is used for items 12 and 19 instead of "risk of bias" (which is more applicable to systematic reviews of interventions) to include and acknowledge the various sources of evidence that may be used in a scoping review (e.g., quantitative and/or qualitative research, expert opinion, and policy document).

Table S2. Search strategy in databases

**PubMed**	("Liver Diseases"[MeSH Terms] OR "hepatitis, viral, human"[MeSH Terms] OR "Hepatitis A"[MeSH Terms] OR "Hepatitis B"[MeSH Terms] OR "Hepatitis C"[MeSH Terms] OR "liver diseases, alcoholic"[MeSH Terms] OR "fatty liver, alcoholic"[MeSH Terms] OR "hepatitis, alco holic"[MeSH Terms] OR "liver cirrhosis, alcoholic"[MeSH Terms] OR "Fibrosis"[MeSH Terms] OR "Non-alcoholic Fatty Liver Disease"[MeSH Terms] OR "Liver Failure"[MeSH Terms] OR "Liver Neoplasms"[MeSH Terms] OR "carcinoma, hepatocellular"[MeSH Terms]) AND ("vaccination/methods"[MeSH Terms] OR "Hepatitis A Vaccines"[MeSH Terms] OR "Hepatitis B Vaccines"[MeSH Terms] OR "Hepatitis C Vaccines"[MeSH Terms] OR "Hepatitis E Vaccines"[MeSH Terms] OR "AIDS Vaccines"[MeSH Terms]) AND ("Randomized Controlled Trial"[Publication Type] OR "Controlled Clinical Tri al"[Publication Type] OR "Random Allocation"[MeSH Terms] OR "Double-Blind Method"[MeSH Terms] OR "Single-Blind Method"[MeSH Terms] OR "Clinical Trial"[Publication Type] OR "Comparative Study"[Publication Type] OR "Evaluation Studies as Topic"[MeSH Terms] OR "Follow-Up Studies"[MeSH Terms] OR "Prospective Studies"[MeSH Terms] OR "Cross-Over Studies"[MeSH Terms])	1993
**Embase**	('liver diseases':ti,ab,kw OR 'viral hepatitis':ti,ab,kw OR 'hepatitis a':ti,ab,kw OR 'hepatitis b':ti,ab,kw OR 'hepatitis c':ti,ab,kw OR 'alcoholic liver disease*':ti,ab,kw OR 'alcoholic fatty liver':ti,ab,kw OR 'alcoholic hepatitis':ti,ab,kw OR 'alcoholic liver cirrhosis':ti,ab,kw OR 'fibrosis':ti,ab,kw OR 'non-alcoholic fatty liver disease':ti,ab,kw OR 'liver failure':ti,ab,kw OR 'liver neoplasms':ti,ab,kw OR 'hepatocellular carcinoma*':ti,ab,kw) AND ('vaccination methods':ti,ab,kw OR 'hepatitis a vaccines':ti,ab,kw OR 'hepatitis b vaccines':ti,ab,kw)	1623
**Scopus**	(TITLE-ABS-KEY ("Active Immunization*" OR "Vaccination* strategi*" OR "Hepatitis A Vaccine*" OR "Hepatovirus Vaccine*" OR "Viral Hepatitis Vaccine*" OR "Hepatitis B Vaccine*" OR "immunization strategy" OR "HIV Vaccine*" OR "AIDS Vaccines") AND TITLE-ABS-KEY ("Liver Disease*" OR "Liver Dysfunction*" OR "Viral Hepatitis" OR "Infectious Hepatitis" OR "Hepatitis A" OR "Hepatitis B" OR "Hepatitis C" OR "Alcoholic Liver Disease*" OR "Alcoholic Fatty Liver" OR "Alcoholic Hepatitis" OR "Alcoholic Liver Cirrhosis" OR Cirrhosis OR "liver health" OR "hepatic disease*" OR "non-viral hepatic disease*" OR "Fatty Liver disease*" OR Fibrosis OR "Liver failure" OR "Liver cancer*" OR "Hepatocellular Carcinoma*") AND TITLE-ABS-KEY ("randomized controlled trial" OR "controlled clinical trial" OR "random allocation" OR "double-blind method")	2610
**WOS**	"Liver Diseases" OR "Viral Hepatitis" OR "Hepatitis A" OR "Hepatitis B" OR "Hepatitis C" OR "Hepatitis E" OR "Alcoholic Liver Disease*" OR "Alcoholic Fatty Liver" OR "Alcoholic Hepatitis" OR "Alcoholic Liver Cirrhosis" OR "Fibrosis" OR "Non-alcoholic Fatty Liver Disease" OR "Liver Failure" OR "Liver Neoplasms" OR "Hepatocellular Carcinoma*" (Topic) and "vaccination methods" OR "Hepatitis A Vaccines" OR "Hepatitis B Vaccines" OR "Viral Hepatitis Vaccines" (Topic)	230

Table S3. Summary of articles reporting characteristics of hepatitis A vaccines in humans, published between 2000 and 2025

Author/Year	Country	Type of Study	Population	Sample Size	Type of Vaccine	Epidemiological Characteristics / Study Focus	Main Findings / Results
Wu et al., 2023 [11]	China	Cross-sectional study	Children under 16 years old in Shanghai (2020–2021) monitored for AEFI with Healive®	1,020	HAVac	Safety assessment of concomitant administration with other vaccines	The concomitant administration of Healive® with other vaccines was found to be safe when compared to administrating Healive® alone.
Wang et al., 2023 [12]	China	Randomized controlled study	Healthy children with negative anti-HAV serum; 300 received Healive®, 100 received Havrix®	400 (375 completed)	HAVac	Immunogenicity comparison between Healive® and Havrix®	The GMCs in the Healive® group were significantly higher than those in the Havrix® group at all time points from 1 to 186 months post-vaccination.
Tricou et al., 2023 [13]	United Kingdom	Randomized controlled study	Healthy adults aged 18–60 years receiving coadministration of HAV vaccine with tetravalent dengue vaccine (TAK-003)	1199 individuals who were screened, 900 were enrolled	HAVac	Evaluation of immunogenicity and safety of HAV vaccine co-administration with dengue vaccine	High DENV seropositivity was achieved within 30 days of a single vaccination. The immune response to one dose of HAV vaccine co-administered with TAK-003 was non-inferior to that of the HAV vaccine alone.
Susarla et al., 2023 [14]	India	Randomized controlled study	Healthy subjects aged 12 months–49 years in two age groups across eight centers	528 (493 completed)	HAVac	Comparison of immunogenicity and safety between Havisure™ and Havrix® hepatitis A vaccines	Both arms achieve 100% seroconversion and seroprotection; no significant difference in GMTs. Common mild AEs included pain, swelling, fever, head- ache. No serious AEs. Havisure™ was safe, immunogenic, and non-inferior to Havrix®.
Thuluva et al., 2021 [15]	India	Randomized controlled study	Healthy HAV vaccine-naïve children aged 1–15 years across eight Indian centers	520 enrolled; 467 completed	HAVac	Comparison of safety and immunogenicity of Indian-made HAPIBEV™ (derived from Healive® bulk) vs Havrix®	Both groups achieved 100% seroconversion, meeting non-inferiority criteria. HAPIBEV™ showed comparable or even higher immunogenicity. The only ad- verse events reported were mild injection site pain.
Susarla et al., 2021 [16]	India	Randomized controlled study	Healthy adolescents and adults aged 12–49 years (two age groups: 12–18 and 19–49)	231 healthy subjects were screened, 55 completed	HAVac	Initial safety and immunogenicity assessment after single dose	Mild AEs (pain, headache, fever); no serious events. 100% seroconversion/seroprotection with marked antibody rise. Safe, highly immunogenic single-dose profile
Ramaswamy et al., 2021 [17]	USA (Alaska)	Cohort study	Alaska Native individuals vaccinated in early childhood; 25-year follow-up	144 enrolled; 43 available at 25 years	HAVac (childhood series)	Assessment of long-term protection and antibody persistence	After 25 years: mean anti-HAV 91.5 mIU/mL; 81.4% protected; model predicts 78.7% protection at 25 years; no booster required.
Chen et al., 2021 [18]	China	Cohort study	Individuals vaccinated with a single dose of HAV-L in childhood; 17-year follow-up	3,515 enrolled, 2,132 followed up	HAV-L	Persistence of anti-HAV antibodies after a single dose	High seroprotection maintained after 17 years, indicating durable long-term immunity without boosters.
Bhave et al., 2021 [19]	India	Cohort study	Indian children vaccinated in 2004 with a single dose of live-attenuated hepatitis A vaccine	143 enrolled; 109 followed up	HAV-L	Anti-HAV antibody persistence 15 years post-vaccination	96% SR in 98 children (no boosters); GMT 79.6 mIU/mL. Overall, 86.2% protection at 15 years; sustained long-term immunity.
Wang et al., 2020 [20]	China	Randomized controlled study	Healthy children vaccinated with Healive® or Havrix® at 0	400 (3:1 randomization)	HAVac	Evaluation of 11-year antibody persistence and prediction	Healive® induced significantly higher GMCs than Havrix® at multiple time points (up to 138 months).

			and 6 months			modeling for long-term protection	Statistical modeling predicted antibody persistence ≥30 years post-vaccination for both vaccines.
Shi et al., 2019 [21]	China	Randomized controlled study	Healthy toddlers, children, and adolescents aged 12 months to 15 years	355 (Group 1: 270; Group 2: 85)	HAVac	Safety evaluation of two-dose primary vaccination 6 months apart	Well-tolerated across age groups. Injection-site reactions: 17.9% vs 33.3%; systemic reactions similar. Unsolicited AEs low; no SAEs. Supports strong pediatric safety profile
Kanik-Yüksek et al., 2019 [22]	Turkey	Retrospective observational study	Children with HAV infection followed in Ankara hospitals, 2008–2015	272 children followed-up	HAVac	Impact assessment of mandatory national vaccination on HAV cases and hospitalization rates	Post-vaccine: HAV cases reduced (72 vs 200 pre-vaccine) with lower hospitalization rates and evidence of herd immunity. No differences in hospitalization indications, complications, or length of stay
Mosites et al., 2018 [23]	USA (Alaska)	Cohort study	143 Alaska Native children aged 3–6 years, seronegative for anti-HAV antibodies, vaccinated under three schedules: 0-1-2, 0-1-6, and 0-1-12 months.	143 enrolled, 46 available for follow-up at 22 years	HAVac	Evaluation of long-term immunogenicity 22 years after vaccination	87% of participants maintained protective anti-HAV antibody levels; supplemental booster doses not needed at 22 years.
Chen et al., 2018 [24]	China	Cohort study	Recipients of single-dose live attenuated HAV vaccine children aged 1–12 years	3,515 enrolled, 51 randomly selected	HAV-L	Assessment of long-term immunity and immune memory 17 years post-vaccination	Robust anamnestic response after booster; persistent HAV-specific memory B/T cells with rapid recall. Long-term immunity confirmed via antibody persistence and cellular memory
Brito et al., 2018 [25]	Brazil	Cross-sectional study	Children aged 2 years vaccinated through National Immunization Program between August 2014 and April 2015	277 enrolled, 265 available	HAVac (single-dose)	Short-term serologic response after single-dose universal vaccination	High seropositivity: 93.6% (236/252) of children tested positive for anti-HAV antibodies. Dried blood spot method reliable for antibody detection. Single-dose vaccination induced strong short-term humoral response.
Zhang et al., 2017 [26]	China	Randomized controlled study	Children aged 18–60 months, HA vaccine-naïve	332 enrolled; 182 with evaluable serum samples	HAVac (single dose)	Evaluation of 5-year antibody persistence and seropositivity	5-year seropositivity: 85.9% (inactivated) vs 90.7% (live). GMCs 76.3 vs 66.8 mIU/mL. No HAV cases. Single dose produced high, durable 5-year seropositivity
Plumb et al., 2017 [27]	USA (Alaska)	Cohort study	Alaska Native children vaccinated at 3–6 years	144 enrolled; 52 available at 20-year follow-up	HAVac, three-dose schedules: 0-1-2, 0-1-6, 0-1-12 months	Antibody persistence and long-term protection 20 years post-vaccination	20-year: 88.5% with ≥20 mIU/mL; GMC 107 mIU/mL. Modeling: GMC 124 at 25 years, 106 at 30 years. Boosters not needed for 25–30 years
Fukushima et al., 2017 [28]	Japan	Cross-sectional study	Healthy Japanese adults (≥20 years) who previously received 2 doses of Aimmugen® Japan between June 2012 and July 2014	20 enrolled	HAVac	Assess immunogenicity of heterologous booster dose for travelers	SR increased from 85% to 100% post-booster. GMT rose from 39.8 mIU/mL to 2938.2 mIU/mL. Third dose of Havrix® effectively boosts immunity and can replace the third Aimmugen® dose.
Espul et al., 2017 [29]	Argentina	Randomized controlled study	Children aged 11–23 months receiving routine HAV vaccination	546 initially; 264 remained at 7-year follow-up	HAVac, one or two doses	Long-term immune response up to 7 years post-vaccination	At 7 years, 204/211 children who received one dose remained seroprotected; all 53 who received two doses remained seroprotected. One or two doses provided strong antibody persistence in toddlers.

Zhang et al., 2016 [30]	China	Randomized controlled study	Third grade children from Jingyuan County were enrolled	956 eligible, 493 randomly selected	HAVac, HAV-L	Comparison of immune per- sistence 12–24 months after a single dose of three different HAV vaccines	Seropositivity at 24 months: H2 63%, Healive® 95.6%, Havrix® 72%, control 1%. GMCs at 24 months: H230.9, Healive® 112.2, Havrix® 44.3 mIU/mL. Healive® showed higher antibody persistence com- pared to H2 and Havrix®. Immunity persisted up to 24 months for all HAV vaccines.
Yu et al., 2016 [31]	China	Randomized controlled study	Healthy children	400 enrolled, 375 participants completed	HAVac 2 doses, 0 & 6 months	Long-term immunogenicity and persistence of antibody response over 5 years	GMCs significantly higher for Healive® than Havrix® at multiple time points. Seroconversion rates reached 100% by 7 months and remained >97% up to 66 months. Predicted duration of protection for Healive®: at least 20 years.
Yoon et al., 2016 [32]	South Korea	Randomized controlled study	Healthy HAV-seronegative adolescents aged 13–19 years	53 enrolled	HAVac 2 doses, 6–12 months apart	Comparison of immunogenicity and safety among three HAV vaccines in adolescents	Seroconversion after 1st dose: 98% (Avaxim™), after 2nd dose: 100% for all. GMCs after 2nd dose: Avaxim™ 7207.7 mIU/mL. Vaccine well-tolerated; no serious AEs.
Spradling et al., 2016 [33]	USA (Alaska)	Prospective cohort study	Alaskan Native children vaccinated for HAV during infancy (6, 12, or 15 months), stratified by maternal anti-HAV status	311 enrolled, 183 followed up	HAVac, 2-dose series	Effect of maternal anti-HAV on persistence of seropositivity through adolescence	Seropositivity ≥20 mIU/mL high through age 10; declined slightly by 15–16 years in some groups. Model predicts ≥30 years persistence in 64% of participants; 84% of those seropositive at 15–16 years predicted to remain seropositive ≥30 years. Supports current infant vaccination recommendations.
Ma et al., 2016 [34]	China	Randomized controlled study	Healthy children aged 18 months to 16 years	6000 (HA-L: 3000 for safety, 251 for immunogenicity; HA-I: 3000 and 2832 for first and booster dose safety, 267 for immunogenicity)	HAV-L and HAVac	Comparison of safety and immunogenicity between live attenuated and inactivated HAV vaccines	Both vaccines were well-tolerated. Seroconversion rates were similar (HA-L 98% vs HA-I 100%). GMCs after first dose similar; after HA-I booster, GMC in- creased significantly (2591.2 mIU/mL). HA-L antigen detected in stool of 25% of tested children. Both vac- cines provide acceptable immunogenicity; further study needed on long-term immunity and potential live vaccine viral shedding.
Theeten et al., 2015 [35]	Belgium	Cohort study	Adults who received 2-dose Havrix™	50 subjects (0,6 months) and 116 subjects (0,12 months)	HAVac	Long-term antibody persistence after 2-dose inactivated HAV vaccination (0–6 or 0– 12-month schedule)	After 20 years, >97% of participants remained seropositive. GMCs were 312 mIU/mL (0–6 months) and 317 mIU/mL (0–12 months). Seven participants lost detectable antibodies but responded strongly to booster. Modeling predicted ≥95% persistence at 30 years and ≥90% at 40 years.
Melgaço et al., 2015 [36]	Brazil	Cross-sectional study	Healthy children/adolescents receiving HAV vaccine	22 enrolled	HAVac, single dose	Evaluated HAV-specific memory cellular response after single dose vaccination com- pared with natural infection	A single dose of inactivated HAV vaccine induced HAV-specific memory B and T cell responses similar to natural infection. Cytokine production (IL-6, IL-10, TNF, IFNγ) increased post-vaccination. Cellular immunity persisted independently of plasma antibody levels, supporting single-dose vaccination as an effective strategy.

Estripeaut et al., 2015 [37]	Panama	Observational study	Children and general population	12,665 viral hep- atitis cases (pop- ulation-level data from surveillance)	HAVac, 2-dose schedule	Evaluated impact of universal 2-dose vaccination on hepatitis A incidence using retrospective and prospective surveillance	Annual incidence of hepatitis A and unspecified hepatitis dropped from 51.1/100,000 pre-vaccination to 3.7–13.1/100,000 post-vaccination. Mean reduction in hepatitis-related outcomes was 82% after vaccine introduction. Herd protection suggested.
Bhave et al., 2015 [38]	India	Cohort study	Healthy children vaccinated at 1–5 years of age	143 enrolled, 121 followed up	HAV-L, single dose	Evaluated long-term immunogenicity 10 years after sin- gle-dose vaccination	98% (106/108) seroprotected; GMT 100.5 mIU/mL. Overall immunogenicity 87.6% (121 children). Single dose provides long-term immunity in Indian children
Lim et al., 2014 [39]	South Korea	Randomized controlled study	Seronegative young adult medical students	582 enrolled, 451 followed up	HAVac, single dose	Evaluated single-dose immunogenicity and influence of demographic characteristics	Overall seroconversion ~11 months: 80.7%. Men: Havrix 81.9% > Epaxal 69.2%; women: Havrix 90.1%, Epaxal 92.9%. Age, obesity, smoking, alcohol had no effect; gender influenced response.
Jain et al., 2014 [40]	India	Randomized controlled study	Healthy children 18–47 months	251 subjects received the first dose: 126 with HAVpur Junior and 125 with Havrix 720 Junior	HAVac, 2 doses, 0 and 6 months	Evaluated non-inferiority, safety, and immunogenicity of a pediatric HAV vaccine in a transitioning endemic setting	SR 1 month post-first dose: 95.9% (HAVpur) vs 96.6% (Havrix). Non-inferiority established. Both vaccines well tolerated with similar ad- verse event profiles. Supports inclusion in childhood immunization programs.
Raczniak et al., 2013 [41]	USA (Alaska)	Prospective cohort	Children vaccinated in childhood (followed into young adulthood)	144 enrolled, 58 in long-term follow-up	HAVac; two-dose vs three-dose at age 3-6 years	Compared duration of protection and antibody persistence between 2- and 3-dose schedules; assessed long-term anti- body persistence up to 17 years	No GMC differences between 2- vs 3-dose schedules at 10–14 years. Protective anti-HAV antibodies persisted ≥14–17 years, plateauing over last 7 years; long-term immunity, no booster needed
Li et al., 2013 [42]	China	Randomized controlled study	Healthy, anti-HAV seronegative toddlers, children, and adolescents	720 (480 AVAXIM, 240 HAVRIX)	HAVac, two-dose schedule (0 and 6 months)	Evaluated safety, immunogenicity, and non-inferiority of AVAXIM vs HAVRIX	AVAXIM 80U Pediatric was non-inferior to HAVRIX 720 for SR at 1-month post-booster; GMTs were higher with AVAXIM; both vaccines were well tolerated with similar AEs
Lolekha et al., 2003 [43]	Thailand	Randomized controlled study	Healthy, seronegative children aged 5–10 years	215 enrolled, 193 followed up	HAVac two-dose schedules with boosters at 6, 12, or 18 months	Evaluated immunogenicity and safety of flexible vaccination schedules	Seroconversion rates >98% in all schedules; equivalent GMC ratios among schedules; large booster response; local reactions <9% after first dose, less frequent after booster; no serious vaccine-related AEs

AE(s) – Adverse event(s); AEFI – Adverse event following immunization; DENV – Dengue virus; GMC – Geometric mean concentration; GMT(s) – Geometric mean titer(s); HAV – Hepatitis A virus; HAVac – Hepatitis A vaccine, HAV-L – Live-attenuated Hepatitis A vaccine; SAE – Serious adverse event; SR – Seroprotection rate.

Table S4. Summary of hepatitis B vaccines in studies reporting seroconversion rates (2000–2025)

Author/Year	Vaccine Type/Formulation	Key Adult Findings	Key Pediatric Findings	Seroconversion Rate (anti-HBs ≥10 mIU/mL)
Yang et al., 2025 [44]	HBVac	High efficacy in the ultra-high responder cohort after second vaccine dose	NA	After third dose: 3/5 volunteers (~60%) had HBsAb >10 mIU/mL, though at low concentrations Four years post-vaccination: 1/5 volunteer (20%) maintained HBsAb >10 mIU/mL
Williams et al., 2025 [45]	HBVac, Heplisav-B	High after booster dose	NA	Standard booster 92.7%, Heplisav-B 99.4%
Vesikari et al., 2023 [46]	HBVac (1A, 3A-HBVac)	3A-HBV: 88.1% after 2.5 year	NA	3A-HBV: 88.1%, 1A-HBV: 72.4%
Furuta et al., 2023 [47]	HBVac (booster)	High	NA	100% seropositivity achieved
Chai et al., 2023 [48]	HBVac	NA	100% after 3 doses	100% seropositivity achieved 11–90 days after full vaccination
Wang et al., 2022 [49]	HBVac + HBIG	NA	MTCT prevention effective	Infants receiving HBIG 100 IU + HBV vaccine: 98.7% Infants receiving HBIG 200 IU + HBV vaccine: 96.7%
Song et al., 2022 †[50]	HBVac	NA	NR	Overall, 93.2% achieved seroprotection; 88.1% in the non-booster group and 99.5% in the booster group.
Peng et al., 2022 [51]	HBVac	Reactivation prevention in adults	NA	At week 0, 61.36% had anti-HBs 10–100 mIU/mL, 22.73% had 100–500 mIU/mL, 2.27% had 500–1000 mIU/mL, and 61.36% had ≥1000 mIU/mL.
Ndububa et al., 2022[52]	HBVac + HBIG	NA	100% MTCT prevention	100% infants HBsAg– at 9 months
Lian et al., 2022 [53]	HBVac	Combination therapy not superior	NA	HBsAg seroconversion at week 48: Experimental group: 3.0% of patients, Control group 2: 1.03% of patients, Control group 1: 1.19% of patients
Cao et al., 2022 [54]	HBVac	64–72% protection vs liver disease	Maternal HBsAg effect: Participants born to HBsAg-positive mothers had significantly higher prevalence of HBsAg, HBeAg, HBeAb, and HBcAb compared to those born to HBsAg-	Vaccination group: 3.79% (2013), 4.66% (2018), 4.50% (2020)

				negative mothers.
Bruce et al., 2022 [55]	HBVac	Long-term protection 86%	Infants & children	Anti-HBs ≥10 mIU/mL 47.3% at 35 y; boosters restore
Zhu et al., 2021 [56]	HBVac (booster)	NA	High in youth	73.8% at 1 year, 67.3% at 4 year<br>Before booster vaccination: Anti-HBs positive rates were 34.50% at 1 year and 73.80% at 4 years after initial vaccination, with an overall rate of 67.32%.<br>After booster vaccination:<br>Age 13–18 years: Anti-HBs positive rate was 47.54% at 4 years, the lowest among all youth age groups.<br>Baseline anti-HBs(-) group: Positive conversion rates were 74.62% at 1 year and 67.66% at 4 years after booster.<br>Baseline anti-HBs(+) group: Positive maintenance rates were 70.16% at 1 year and 66.66% at 4 years after booster.
Zhou et al., 2021 [57]	HBVac	NA	MTCT reduced	Among infants in the PVST program:<br>Anti-HBs positive rate: 96.84%<br>HBsAg positive rate: 0.77%<br>Both negative: 2.39%<br>Among 15 infants with initially double-negative results who completed revaccination and PVST again, 14 (93.33%) became anti-HBs positive.
Zhang et al., 2021 [58]	HBVac + HBIG	NA	High dose improved anti-HBs	Maternal HBV DNA < 5 log10 IU/mL<br>10 µg HB vaccine: 77.8% (High + Very high responders: 54.9% + 22.9%)<br>20 µg HB vaccine: 87.6% (High + Very high responders: 45.4% + 42.2%)<br>Maternal HBV DNA ≥ 5 log10 IU/mL<br>10 µg HB vaccine: 78.1% (High + Very high responders: 48.4% + 29.7%)<br>20 µg HB vaccine: 85.4% (High + Very high responders: 54.9% + 30.5%)
Thompson et al., 2021 [59]	HBVac	Not reported	Birth-dose MTCT prevention	Not reported Seroconversion Rate<br>Birth-dose hepatitis B vaccination: 68% of infants received a birth-dose vaccine, and 77% of those were administered timely.<br>HBV mother-to-child transmission (MTCT): No cases were observed, indicating effective protection.
Miyakawa et al., 2021 [60]	HBVac	Not reported	Child infection 1.9%	Overall seroprotection in children: 84.8% (i.e., 100% – 15.2% below seroprotective level)

				By subgroups (approximate, based on aOR analysis):<br>Complete HepB vaccination: high seroprotection (reference group)<br>No or incomplete HepB vaccination: lower seroprotection<br>HBIG at birth: variable, but some reduction in seroprotection
Li et al., 2021 [61]	HBVac	Protective immunity persisted ~11 year	NA	2008 (28 days after 3rd dose, N = 428): 411/428 → 96.0% seropositive<br>2014 (6 years after 3rd dose, N = 276): 133/276 → 48.2% seropositive<br>2019 (11 years after 3rd dose, N = 239): 97/239 → 40.6% seropositive
Huang et al., 2021 [62]	HBVac	NA	Non-response 1.7%	At different PVST intervals:<br>1– month: 92.1%<br>2– month: 94.3%<br>3– month: 96.5%<br>4– month: 95.6%<br>5– month: 93.3%<br>6– month: 97.5%<br>7–8 months: 97.4%
Chowdhury et al., 2021 [63]	HBVac (LOCAL HBVac, Engerix-B vaccine)	NA	Not reported	Day 30, prior to 2nd dose): Hepa-B 5.33%, Engerix-B 6.85%<br>Day 60, following 1st dose): Hepa-B 86.67%, Engerix-B 69.86%<br>Day 180, prior to 3rd dose): Hepa-B 96%, Engerix-B 90.41%<br>Day 210, following 1st dose): Hepa-B 98.67%, Engerix-B 100%
Atsmon et al., 2021 [64]	HBVac	98.8–100%	Not reported	Month 1: Early detection of HBsAb; high responders (anti-HBs ≥100 mIU/mL) 36.4%<br>Month 3 (two months after second dose): 98.8% seroprotected (95% CI: 93.7–99.7%)<br>Month 7: 100% seroprotection; high responders 97.6%
Ren et al., 2020 [65]	HBVac	0-1-3-month schedule better	Not reported	Group I: vaccinated at 0–1–3 months; group II: vaccinated at 0–1–6 months; group III: vaccinated at 0–1–12 months.<br>1 month after vaccination:<br>Group I (0–1–3 months): 77.6%<br>Group II (0–1–6 months): 86.9%<br>Group III (0–1–12 months): 92.9%<br>8 years after vaccination:<br>Group I: 68.3%<br>Group II: 57.8%<br>Group III: 47.3%
Raven et al., 2020 [66]	HBVac	67–87%	Not reported	Seroprotection (response) rates at 3 months after revaccination in vaccine non-responders:<br>Control (repeated initial vaccination): 67% (83/124, 95% CI 57.9–75.1)<br>Twinrix 20 µg: 80% (94/118, 95% CI 71.3–86.5)

				HBVaxPro 40 μg: 83% (95/114, 95% CI 75.2–89.7)<br>Fendrix 20 μg: 87% (108/124, 95% CI 79.9–92.4)
Qiu et al., 2020 [67]	HBVac	NA	Robust persistence 8 year post-booster	Seroprotection rates after booster hepatitis B vaccination in children:<br>Group II (10 μg HBV, 0-1-6-month schedule): 92.8% (1 month after dose 1), 99.7% (1 month after dose 3), 97.6% (1 year), 90.3% (5 years), 83.4% (8 years)<br>Group C (pre-booster anti-HBs 1 to <10 mIU/mL): 98.9% (1 month after dose 1), 99.9% (1 month after dose 3), 99.5% (1 year), 95.5% (5 years), 92.8% (8 years)
Li et al., 2020 [68]	HBVac	Not reported	Higher dose improved anti-HBs	Seroprotection (anti-HBs) data from the study on infants born to HBsAg-positive mothers with high viral load:<br>7 months: 10 μg group: 652.48 mIU/mL vs 20 μg group: 1541.72 mIU/mL<br>12 months: 10 μg group: 257.44 mIU/mL vs 20 μg group: 1073.41 mIU/mL
Insulander et al., 2020 [69]	HBVac	NA	82% protective at 8–12 yr	Seroprotection (anti-HBs) data from the long-term follow-up study:<br>13 months: Very high percentage of infants had protective anti-HBs levels (exact % not specified).<br>8–12 years: 56/68 children (82%) maintained protective anti-HBs levels.<br>After one booster dose: Anti-HBs levels in previously low responders (<10 IU/L) increased to protective levels.
Ford et al., 2020 [70]	HBVac	Not reported	NA	100%
Dhouib et al., 2020 [71]	HBVac	Not reported	Birth-dose 99.4% effective	Hepatitis B birth-dose vaccination (HepB-BD) achieved 99.4% protection against hepatitis B infection, compared with 77% protection when the first dose was given at 3 months.
Cabezas et al., 2020 [72]	HBVac	Not reported	Immunity improved	Seroprotection: Twenty-three years after introduction of hepatitis B vaccination in Abancay, Peru, 66.36% of individuals negative for HBsAg and anti-HBc had protective anti-HBs levels (≥10 mIU/mL).
Zhao et al., 2019 [73]	HBVac	Strong booster response	Not reported	Seventeen to twenty years after primary hepatitis B vaccination, 74.5% of participants retained protective anti-HBs levels (≥10 mIU/mL), which increased to 93.1% achieving seroprotection after a single booster dose.
Van Mulder et al., 2019 [74]	HBVac	Higher response than IM	Not reported	Following booster vaccination, 100% of participants achieved seroprotective anti-HBs responses, with all subjects showing an anamnestic response by day 14 that persisted through day 210.
Van Damme et	HBVac	20–30-year protection	Not reported	Anti-HBs seroprotection (≥10 mIU/mL) was present in 90.1% of

al., 2019 [75]				participants pre-challenge and reached 100% by 30 days after the challenge dose.
Tfifha et al., 2019 [76]	HBVac 4-dose	Not reported	77.2%	The seroprotection rate (anti-HBs ≥10 IU/L) was 77.2% among children under 6 years old after the four-dose primary hepatitis B vaccination.
Lu et al., 2019 [77]	HBVac 3-dose vs 2-dose	Not reported	NA	At 12-year follow-up, the seroprotection rate (anti-HBs ≥10 mIU/mL) was 71.78% in adults vaccinated on the three-dose (0–1–6 month) schedule and 53.61% in those vaccinated on the two-dose (0–6 month) schedule.
Lee et al., 2019 [78]	HBVac (monovalent and combination hepatitis B vaccine)	Not reported	Low prophylaxis failure	Both regimens achieved high and comparable seroprotection, with similar anti-HBs antibody levels at 9 months of age (mean anti-HBs 643 ± 374 IU/L for monovalent vs 561 ± 396 IU/L for combination vaccine), indicating effective protective immunity in infants born to HBsAg-positive mothers.
Giao et al., 2019 [79]	HBVac	Not reported	34% timely completion	Not reported
Zhou et al., 2018 [80]	HBsAg-HBIG therapeutic	Cytokine predictors of response	Not reported	Not reported
Zhang et al., 2018 [81]	HBVac	HBBI 5.36%; younger adults higher	Not reported	10–99 mIU/mL: 79/252=31.35%<br>100–999 mIU/mL: 72/252=28.57%<br>≥1000 mIU/mL: 39/252=15.48%<br>Overall seroprotection rate: 75.4%.
Wu et al., 2018 [82]	HBVac booster	Not reported	High seroprotection	Group I (<10 mIU/mL, 3-dose booster) – 95.65% (1 month after dose 1), 99.67% (1 mo after dose 3), 97.59% (1 yr after dose 3), 91.05% (5 yr after dose 3); Group II (≥10 mIU/mL, 1-dose booster) – 100% (1 mo after dose 1), 99.87% (1 mo after dose 3), 99.66% (1 yr after dose 3), 98.21% (5 yr after dose 3).
Weinberger et al., 2018 [83]	HBVac	Elderly impaired primary response	Not reported	Primary series: 100% seroprotection in young adults; ~71% in older adults.<br>Booster dose: Seroprotection restored in almost all participants regardless of age, demonstrating effective immune memory.
Wei et al., 2018 [84]	HBVac + HBIG	Not reported	MTCT prevention effective	Positive seroprotection (anti-HBs) after HBIG + HepB in infants born to HBsAg-positive mothers:<br>At 7 months:<br>100 IU HBIG + HepB: 98.5% (529/537)<br>200 IU HBIG + HepB: 98.2% (609/620)<br>At 12 months:<br>100 IU HBIG + HepB: 98.2% (431/439)

				200 IU HBIG + HepB: 97.1% (496/511)
Wang et al., 2018 [85]	HBVac 20 μg 3-dose	98.3%	NA	Positive seroprotection (anti-HBs) 2 years after primary hepatitis B vaccination in healthy young adults:<br>Group A: 20 μg, 0-1-6 month → 98.31% seroprotection<br>Group B: 60 μg, 0-1 month → 88.37% seroprotection<br>Group C: 60 μg, 0-2 month → 85.19% seroprotection
Saffar et al., 2018 [86]	HBVac neonatal	~90% long-term protection	Youth 18–20 y	Nearly 90% maintained long-term protective anti-HBs levels without requiring a booster
Pronocitro et al., 2018 [87]	HBVac	Not reported	73.1%	73.1%
Kishino et al., 2018 [88]	HBVac (mpHBV)	Non-inferior to Heptavax	NA	Seroprotection (anti-HBs ≥10 mIU/mL) at 1-month postdose 3 (Month 7):<br>mpHBV SC: non-inferior to Heptavax®-II SC<br>mpHBV IM: higher anti-HBs geometric mean titers than SC groups<br>Safety: Most local/systemic adverse events mild-to-moderate; no serious vaccine-related AEs
Jackson et al., 2018 [89]	HBVac (HEPLISAV-B™, [Engerix-B®)	Superior seroprotection	Not reported	Seroprotection (anti-HBs ≥10 mIU/mL) at week 28:<br>Diabetes participants: 90.0% (HBsAg-1018) vs. 65.1% (HBsAg-Eng)<br>Overall population: HBsAg-1018 group had statistically significantly higher seroprotection than HBsAg-Eng
Hyer et al., 2018 [90]	HBVac	Improved immunogenicity	Not reported	Not reported
Zhu et al., 2017 [91]	HBVac (Hepavax-Gene TF)	MTCT prevention >95%	Neonates	Mother-to-child HBV transmission was prevented in >95% of neonates vaccinated with Hepavax-Gene TF in HBV-positive mothers, noninferior to Engerix-B.<br>>90% of neonates achieved seroprotective antibody levels at 1 and 6 months post vaccination for Hepavax-Gene TF.
Wang et al., 2017 [92]	HBVac	Non-response 6.7% adults	Infants 6.7%	Seroprotection rate (anti-HBs ≥10 mIU/mL) after standard hepatitis B vaccination: 93.3% (306/328 infants)<br>Non-response rate (anti-HBs <10 mIU/mL): 6.7% (22/328 infants)<br>By HBsAg status at birth:<br>HBsAg-positive infants: 87.0% seroprotected (non-response 13.0%)<br>HBsAg-negative infants: 95.0% seroprotected (non-response 5.0%)
Verso et al., 2017 [93]	HBVac	Low responders common	Students	Overall seroprotection rate (anti-HBs ≥10 mIU/mL): 61.9%<br>Non-seroprotected (anti-HBs <10 mIU/mL): 38.1%
Tan et al., 2017 [94]	HBVac 3–4 dose	Not reported	Preterm infants 95.5%	Seroprotection rate (anti-HBs ≥10 IU/L): 95.5% (22/23 surviving infants)<br>Non-seroprotected: 4.5% (1/23 infants)

				HBsAg positivity: 0% (all infants were negative)
Tai et al., 2017 [95]	HBVac 3-dose	Lower seroconversion in failures	Children HBeAg+	Vaccine failure linked to genotype C, maternal HBsAg
Pinto et al., 2017 [96]	HBVac infant 3-dose	Strong booster response	Adolescents	After infant vaccination, residual anti-HBs was low in adolescence, but immune memory remained robust, with ≥91% achieving seroprotective levels after booster challenge. A small proportion lost immune memory (2.2% in 10–11 y, 5.6% in 15–16 y).
Lu et al., 2017 [97]	HBVac ± HBIG	Not reported	Neonates >97% anti-HBs	Anti-HBs seroprotection (≥10 mIU/mL) at 7 months:<br>Vaccine alone: 129/132 (97.7%)<br>Vaccine + HBIG: 740/751 (98.5%)<br>Anti-HBs seroprotection at 12 months:<br>Vaccine alone: 596/612 (97.4%)<br>Vaccine + HBIG: 118/120 (98.3%)
Lu et al., 2017 [98]	HBVac 3-dose booster	Not reported	Children 5–15 years 93.8–100%	A 3-dose booster series effectively restored protective anti-HBs levels in previously vaccinated children, with up to 100% seroprotection after the third dose and persistence above 73% at 5 years.
Lee et al., 2017 [99]	HBVac	Not reported	Children 96% after booster	72 HBsAb-negative children received a single booster dose.<br>96% (69/72) developed protective antibodies (seroprotection ≥10 IU/L)<br>4% (3/72) remained non-protected
Huynh et al., 2017 [100]	HBVac	Not reported	Children/adolescents	Seroprotection (anti-HBs ≥10 IU/L): <10% in 5–19-year-olds in the vaccinated cohort.
Wang et al., 2016 [101]	HBVac 60 mg	Safe	Not reported	Seroprotection rates (anti-HBs ≥10 mIU/mL) in adults after vaccination:<br>Group A (20 μg Engerix-B, 0-1-6 month): 93.17%<br>Group B (20 μg Kangtai, 0-1-6 month): 97.23%<br>Group C (60 μg Kangtai, 0-2 month, rapid schedule): 93.54%<br>Group D (20 μg Huabei, 0-1-6 month): 98.98%
Van Der Meeren et al., 2016 [102]	HBVac	65.4% pre-challenge; 97.9% post-challenge	Adolescents	Seroprotection rates (anti-HBs ≥10 mIU/mL) in adolescents 15–16 years after infant vaccination (3-dose series):<br>Pre-challenge: 65.4% (95% CI: 59.6–70.9%)<br>1-month post-challenge (booster dose): 97.9% (95% CI: 95.6–99.2%)
Klushkina et al., 2016 [103]	HBVac	Acute HB sharply reduced	General population	While specific seroprotection (anti-HBs ≥10 mIU/mL) was not measured, the very low HBsAg prevalence in vaccinated children indicates effective population-level protection from hepatitis B.
Katoonzadeh et al., 2016 [104]	HBVac	Declined with age	Children/adolescents	Seroprotection (anti-HBs ≥10 IU/mL) after 10–18 years post-primary vaccination:<br>10–11 years old: 48% seroprotected

				12–14 years old: intermediate (not explicitly reported but lower than youngest group)<br>15–18 years old: 26.5% seroprotected
Hsu et al., 2015 [105]	HBVac	Not reported	Children <18 y	Not reported
Qu et al., 2014 [106]	HBVac (3-dose neonatal series)	NA	Long-term efficacy of neo- natal HBV vaccination	Neonatal vaccination: HBsAg seroprevalence reduction 72% (95% CI 68–75%) in early adulthood;<br>Catch-up vaccination at 10–14 years: HBsAg seroprevalence reduction 21% (95% CI 10–30%);<br>Booster at 10–14 years for infants born to HBsAg-positive mothers: hazard ratio 0.68 (95% CI 0.47–0.97) for HBsAg positivity.
Pan et al., 2014 [107]	HBVac with high antigen content	safety of high-dose	NA	60 μg HB vaccine (high antigen): seroconversion ≥92.1% from the second dose; GMC ≥286.0 mIU/mL<br>30 μg HB vaccine: seroconversion ≥87.1% from the second dose; GMC ≥164.0 mIU/mL<br>10 μg HB vaccine (standard): seroconversion ≥83.0% from the second dose; GMC ≥110.1 mIU/mL

† Study reports vaccine breakthrough infections (VBIs) despite prior seroprotection. Abbreviations used are: HBV, Hepatitis B virus; HBsAg, Hepatitis B surface antigen; HBeAg, Hepatitis B e antigen; HBIG, Hepatitis B immunoglobulin; HBVac, Hepatitis B vaccine; IM, Intramuscular; mpHBV, Modified-process hepatitis B vaccine; MTCT, Mother-to-child transmission; NA, Not applicable; PVST, Post-vaccination serologic testing; POCT, Point-of-care testing; OBI, Occult hepatitis B virus infection; SC, Subcutaneous.

Table S5. Summary of studies evaluating hepatitis B virus vaccination, serologic response, and immune outcomes, published between 2000 and 2025

Author/Year	Country	Type of Study	Population	Sample Size	Type of Vaccine	Epidemiological Characteristics / Study Focus	Main Findings / Results
Yang et al., 2025 [44]	China	Observational study	Healthy adult volunteers (ultra-high and extremely low responders to HBV vaccination)	49 enrolled, 44 followed up	HBVac	Examined persistence of HBsAb levels and IgG-H CDR3 repertoire characteristics among individuals with extremely high vs. low antibody responses after vaccination	Ultra-high responders showed peak HBsAb levels (mean≈25,000 mIU/mL) after the 2nd dose, gradually declining over 4 years. Distinct IGHV gene usage and CDR3 motifs were associated with sustained antibody levels. Findings suggest IGHV mutation and repertoire features influence long-term vaccine response.
Williams et al., 2025 [45]	USA	Cohort study	Health care workers with prior HBV vaccination but anti-HBs <10 mIU/mL (2019–2022); KPSC members ≥18 years who received ≥1 HepB-CpG or HepB-alum dose at KPSC clinics (Aug 2018–Nov 2020)	693 enrolled, 242 followed up	HBVac	Compared seroprotection rates between Heplisav-B and standard HBV vaccine as single boosters in previously immunized, low-titer health care workers	Seropositivity achieved in 92.7% of standard booster group and 99.4% of Heplisav-B group. Both vaccines effective, but Heplisav-B produced slightly higher antibody response.
Vesikari et al., 2023 [46]	Finland	Cohort study	Adults previously vaccinated and seroprotected in a prior phase 3 trial	465 (244 received 3A-HBV; 221 received 1A-HBV)	HBVac	Evaluated persistence of protective antibody levels (anti-HBs ≥10 mIU/mL) 2.5 years after vaccination with 3-antigen vs. single-antigen HBV vaccine	After 2.5 years, seroprotection persisted in 88.1% of 3A-HBV vs. 72.4% of 1A-HBV recipients ($p < 0.0001$). Mean anti-HBs titers significantly higher in 3A-HBV group. Stronger initial response predicted sustained protection.
Furuta et al., 2023 [47]	Japan	Feasibility study	Healthy adult blood center personnel previously boosted with HBV vaccine	3 enrolled	HBVac	Explored the development of recombinant HBIG using B cells from vaccinated donors to create HBV-neutralizing monoclonal antibodies	Successfully produced recombinant monoclonal antibodies with strong HBV-neutralizing activity, exceeding current HBIG potency. Antibodies bound HBsAg conformational epitopes without cross-reactivity to human molecules, indicating feasibility for alternative HBIG production.
Chai et al., 2023 [48]	China	Observational study	Hospitalized children with documented HBV vaccination history	6736 enrolled, (1,638 one dose, 1,167 two doses, and 3,931 full dose	HBVac	Analyzed HBsAb positivity rate and antibody levels over time after each of the three vaccine doses in children	HBsAb positivity and geometric mean concentration increased with each dose, peaking after the third dose. 100% seropositivity achieved 11–90 days after full vaccination; antibody levels remained >90% and >100 mIU/mL for one year, then gradually declined over 9–10 years. Non-response rate estimated <5% with appropriate testing time.
Wang et al., 2022 [49]	China	Randomized comparative study	HBsAg- and HBeAg-positive pregnant women and their newborns	331 mother–infant pairs	HBVac + HBIG 100 IU vs. 200 IU	Compared efficacy of two HBIG dosages combined with HBV vaccine in preventing MTCT of HBV	MTCT rates did not differ significantly between 100 IU and 200 IU HBIG groups ($p > 0.05$). High maternal HBV DNA load was a major risk factor for immunoprophylaxis failure. A single 100 IU HBIG dose with HBV vaccine achieved effective MTCT prevention and was more cost-efficient.

Song et al., 2022 [50]	China	Cohort study	Children born to mothers with chronic HBV infection (HBsAg-positive)	454 children	HBVac	Evaluated long-term protection, vaccine breakthrough infections (VBIs), and booster effect among infants born to HBsAg-positive mothers	HBsAb declined 7 mo–2 y; 6.8% (31/454) had VBIs (7 overt, 7 occult). Risk factors: maternal HBeAg+, high HBV DNA/HBsAg, low infant anti-HBs, no booster. VBI: 0.5% boosted vs 11.9% non-boosted (p<0.001); no boosted child developed infection
Peng et al., 2022 [51]	China	Randomized con- trolled study	Patients with occult hepatitis B virus infection (OBI), 20 µg per dose, 0–4–24 weeks schedule	200 enrolled; 44 in vaccine group (Group A), 39 in control group (Group B) after exclusions 1,578-person cohort	HBVac	Investigated immune responses and therapeutic potential of HepB vaccination in OBI patients HepB vaccination and response to booster dose	At baseline, ~84% of patients were HBsAb positive with low antibody levels. At week 36, all Group A patients were anti-HBs positive with median 1000 mIU/ml, significantly higher than baseline and Group B (p<0.05). CD8+ T and B lymphocyte counts increased significantly in Group A compared with Group B. Two patients in control group showed HBV reactivation. HBsAb levels were positively correlated with CD8+ T and B lymphocytes over time.
Ndububa et al., 2022 [52]	Nigeria	Cohort study	Pregnant women screened for HBV infection and their infants	10,866 pregnant women; 395 with chronic HBV infection; 376 completed follow-ups	HBVac + HBIG	Assessed prevention of MTCT of HBV and out- comes of exposed infants after 9 months	HBV prevalence among pregnant women was 3.64%. 5.2% were HBeAg positive; 17 women received TDF. 260 newborns received HepB + HBIG; others received HepB alone. All infants tested negative for HBsAg at 9 months. Mean maternal age 31.5 years; perinatal mortality rate 29.2/1000 births. Study showed complete prevention of HBV MTCT with vaccination and HBIG.
Lian et al., 2022 [53]	China	Randomized con- trolled study	Treatment-naïve HBeAg-positive chronic hepatitis B patients, multicenter from nine liver centers	303 patients	HBVac	Evaluated efficacy and safety of pegylated interferon-α-2b plus TDF, GM-CSF, and hepatitis B vaccine versus standard treatments	Week 48 HBsAg seroconversion: 3.0% (experimental), 1.03% (TDF+peg-IFN), 1.19% (IFN); NS. Greater HBsAg decline in experimental & TDF+peg-IFN vs IFN (p=0.008, 0.006); no difference between experimental & TDF+peg-IFN (p=0.619). AEs similar; lower neutropenia in experimental. Combination not superior to TDF+peg-IFN.
Cao et al., 2022 [54]	China	Randomized con- trolled trial	Individuals from Qidong, China, followed from infancy, administered at birth, 1, and 6 months	82,866 par- ticipants (41,136 vaccinated; 41,730 controls)	HBVac	Evaluated 37-year long-term efficacy of hepatitis B vaccination in preventing liver cancer and liver disease mortality	Liver cancer incidence significantly lower in vaccinated group (HR 0.28; 95% CI 0.11–0.70; p = 0.007). Vaccine efficacy: 72% protection against liver cancer, 70% against liver cancer deaths, and 64% against deaths from liver diseases. Hepatitis B vaccination at birth provided strong long-term protection.
Bruce et al., 2022 [55]	USA (Alaska)	Cohort study	Alaska Native adults and children vaccinated against HBV	320 participants from original 1,578-person cohort	HBVac	Assessed antibody persistence and immune protection 35 years after primary HepB vaccination and response to booster dose	After 35 years, 47.3% of participants maintained anti-HBs ≥10 mIU/ml. Among those with low antibodies who received a booster, 73.7% responded with HBsAb ≥10 mIU/ml at 30 days. Estimated 86% overall retained protection 35 years post-vaccination. No HBV surface antigen or DNA detected in any participant. Booster doses deemed unnecessary for general population.
Zhu et al., 2021 [56]	China	Observational study	Youth who received hepatitis B booster vaccination	37,788 participants	HBVac (booster dose)	Evaluated positive rates and persistence of HBsAb after booster vaccination over 4 years	HBsAb+ rates: 34.5% pre-booster, 73.8% at 1 y, 67.3% at 4 y. Ages 13–18 had lowest 4-y positivity (47.5%). Baseline HBsAb–: 74.6% → 67.7%; baseline HBsAb+: 70.2% → 66.7%. Positivity declined over time post-booster.

Zhou et al., 2021 [57]	China	Observational study	HBsAg-positive mothers and their infants	2,120 mother–infant pairs	HBVac	Evaluated implementation and influencing factors of PVST to prevent mother-to-child HBV transmission	PVST participation 67.1%. Infants: HBsAg+ 0.77%, anti-HBs+ 96.8%, double-negative 2.39%; 93.3% of double-negative seroconverted after revaccination. Higher maternal education, older age, and local residence ↑ follow-up. PVST + revaccination improved HBV protection
Zhang et al., 2021 [58]	China	Cohort study	Infants born to HBsAg-positive mothers, multicenter	955 mother–infant pairs	HBVac (10 µg vs. 20 µg) + HBIG	Evaluated impact of higher HB vaccine dose on MTCT prevention and immune response	At 12 mo, 13 infants HBsAg+. 20 µg vs 10 µg: no significant MTCT reduction, but ↑ high anti-HBs (≥1000 IU/L) and ↓ low-response in infants of mothers with HBV DNA <5 log_{10} IU/mL. No safety concerns
Thompson et al., 2021 [59]	Democratic Republic of the Congo	Feasibility study	Pregnant women and their infants screened for HBV through HIV PMTCT infrastructure	90 enrolled, 88 infants followed up	HBVac	Birth-dose vaccination into existing HIV PMTCT program	90 women enrolled; 88 live-born infants. 68% received birth-dose HBV vaccine, 77% within 24 h. Facility-born: 76% vaccinated, 85% timely; outside: 48%, 42% timely. No AEs. At 24 wk, all 53 tested infants HBsAg–; MTCT fully prevented
Miyakawa et al., 2021 [60]	Vietnam	Cohort study	Pregnant women and their children	1987 mother–child pairs (1339 children followed up)	HBVac	Evaluated HBV seroprevalence and transmission risk factors after universal vaccination introduction	12.6% of mothers were HBsAg-positive. At 2-year follow-up, 1.9% of children were HBsAg-positive; 28.3% infection among children of HBeAg-positive mothers despite full vaccination. Low maternal BMI and incomplete HepB doses were associated with child infection or low antibody levels. HBV infection rate in children remained low overall.
Li et al., 2021 [61]	China	Cross-sectional study	Adult non-responders to routine HB vaccination, 3-dose schedule: 0, 28, 56 days	428 in immunogenicity analysis; 276 followed in 2014; 239 in 2019	HBVac	Evaluated long-term immune persistence after high-dose HepB revaccination in previous non-responders	Seropositive rate decreased from 96% post-vaccination to 48.2% (2014) and 40.6% (2019). HBsAb GMC declined over time but remained protective in many. 24.9% showed increased antibody titers in 2019, suggesting natural boosting from HBV exposure. Protective immunity persisted for about 11 years without need for further boosters.
Huang et al., 2021 [62]	China	Cohort study	Infants born to HBsAg-positive mothers, 3-dose schedule: 0, 1, 6 months	1,255 infants	HBVac	Determined optimal timing for PVST	Overall non-response 1.7%. Higher when PVST at 7–8 mo (5.7%) vs 1 mo (1.6%). Anti-HBs titers declined with longer intervals. Optimal PVST: 7 mo or 1 mo post-final dose
Chowdhury et al., 2021 [63]	Bangladesh	Randomized clinical trial	Healthy adults, three-dose schedule: 0, 1, 6 months	158 participants	HBVac	Compared immunogenicity and safety of local Hepa-B vaccine to Engerix-B	Seroconversion rates were 98.67% (Hepa-B) and 100% (Engerix-B). Geometric mean titer ratios exceeded 0.5 non-inferiority margin at all time points. Mild injection site soreness was common; no serious AEs occurred. Hepa-B proved non-inferior to Engerix-B in efficacy and safety.
Atsmon et al., 2021 [64]	Israel	Randomized clinical trial	Healthy adults aged 20–40 years	91 participants	HBVac	Evaluated immunogenicity and safety to qualify new reference standard batch	Seroprotection rate reached 98.8% by month 3 and 100% by month 7. High responders were 81.4% at month 3 and 97.6% at month 7. Geo- metric mean concentration rose from 413.6 mIU/mL (month 3) to 6799.9 mIU/mL (month 7). Vaccine was safe and well-tolerated with no serious AEs.
Ren et al., 2020 [65]	China	Cohort study	Healthy adults negative for HBsAg, anti-HBs, and HBsAb between 2011-2018	777 enrolled, 242 followed up	HBVac	Evaluated long-term persistence of anti-HBs and compared three vaccination schedules (0-1-3, 0-1-6, 0-1-12 months) over 8 years	Seroprotection rate declined from 85.9% at 1-month post-vaccination to 58.3% after 8 years. GMT dropped from 158.49 to 15.14 mIU/mL. The 0-1-3-month schedule showed similar or better long-term persistence than 0-1-6 month, and better than 0-1-12-month schedule. No correlation between early and long-term HBsAb titers was observed.

Raven et al., 2020 [66]	Nether- lands	Randomized clinical trial	Healthy adults (18–80 years) who were non-responders to primary hepatitis B vaccination	480 enrolled and randomly as- signed	HBVac	Compared serological response rates among different revaccination regimens in healthy non-responders	At month 3, responder rates were 67% in control, 83% in HBVaxPro-40, and 87% in Fendrix groups. HBVaxPro-40 and Fendrix showed significantly higher responder rates than control (p=0.0204 and p=0.0006, respectively). Only one serious AE (herpes zoster ophthalmicus) occurred, unrelated to vaccination.
Qiu et al., 2020 [67]	China	Cohort study	Children aged 5–15 years previously vaccinated against HBV in infancy	4170 (2326 negative for all HBV markers)	HBVac (5 µg, 10 µg, 20 µg)	Evaluated immune persistence and booster strategies among children with different pre-booster anti-HBs levels	Group II (10 µg) seropositive 92.8%–83.4%; GMT 4194.5 mIU/mL post-first dose, 122.6 mIU/mL at 8 y. Pre-booster anti-HBs 1–<10 mIU/mL → stronger, longer-lasting response (p<0.001). Robust persistence up to 8 years post-booster
Li et al., 2020 [68]	China	Cohort study	Infants born to HBsAg-positive mothers with HBV DNA >6 $\log_{10}$ IU/mL	549 infants (349 received 10 µg, 200 received 20 µg HepB)	HBVac (10 µg vs 20 µg per dose)	Evaluated effect of in- creased vaccine dose on anti-HBs response and oc- cult HBV infection (OBI) rates	HBsAb levels were significantly higher in 20 µg group at 7 and 12 months. OBI incidence was lower in 20 µg group (7.56–6.9%) vs 10 µg group (21.55–17.07%). Infants with HBsAb < 100 mIU/mL had higher OBI risk. Increasing vaccine dose to 20 µg improved antibody response and reduced OBI in high-risk infants.
Insulander et al., 2020 [69]	Sweden	Cross-sectional study	Infants born to HBsAg-positive mothers, follow-up at 8–12 years, at birth and 1 month; hexavalent vaccine (including HBV) at 3, 5, and 12 months	68 children	HBVac	Evaluated long-term persistence of anti-HBs and infection prevention after infant vaccination program	At 8–12 years, 82% maintained protective anti-HBs levels; 2 children showed anti-HBc seroconversion without disease; none had active infection. Booster restored immunity in all children with low titers. Vaccination program provided long-term protection and effective immune memory.
Ford et al., 2020 [70]	Argentina	Prospective study	Adult patients attending emergency department between April and May of 2018	100 patients (75 POCT, 25 laboratory)	HBVac	Evaluated feasibility of HBV screening and vaccination in an emergency department in a resource-limited setting	No patients tested positive for HBsAg. All who completed vaccination were screened using POCT. None screened by laboratory returned for vaccination. Same-day vaccination improved completion rate (75% vs. 14%, p<.001). POCT-based screening with immediate vaccination increased vaccine uptake in ED setting.
Dhouib et al., 2020 [71]	Tunisia	Cohort study	Children receiving hepatitis B vaccination (birth dose vs. 3-month dose) during 2000 to 2017	1526 participants	HBVac (mono-valent, birth or 3-month)	Compared efficacy of first HBV vaccine dose given at birth versus at 3 months and assessed long-term vaccine impact	AR was 5.67/100,000 PY for 3-month protocol vs. 0.11/100,000 PY for birth dose. RRR was 77% for 3-month and 99.4% for birth dose. No HB cases in children aged 5–11 under birth-dose protocol. Annual HB cases dropped from 112 (2000) to 48 (2017). Birth dose highly effective (99.4%) in preventing HBV infection.
Cabezas et al., 2020 [72]	Peru	Cross-sectional study	General population aged 0–94 years from an HBV/HDV hyperendemic region (Abancay) be- tween November and December 2014	3,165 participants	HBVac	Evaluated HBV and HDV prevalence 23 years after the first pilot HepB vaccination program	HBsAg prevalence dropped to 1.2%, anti-HBc to 41.7%, and anti-HBs ≥10 mIU/mL in 66.4% of HBV-negative individuals. No HBsAg detected in children <15 years; HDV antibodies found only in 5.3% of adult carriers. HBV endemicity declined from high to low; HDV eliminated among those <30 years.
Zhao et al., 2019 [73]	China	Cohort study	Young adults born 1997–1999 who received full HepB (CHO) vaccination in infancy	1,352 participants	HBVac (CHO-derived)	Evaluated immune persistence 17–20 years post-primary vaccination and response to booster dose	74.5% retained anti-HBs ≥10 mIU/mL after 17–20 years (GMC 57.4 mIU/mL); HBsAg carrier rate 0.4%. After booster, 93.1% with initially low anti-HBs responded with anti-HBs ≥10 mIU/mL (GMC 368.7 mIU/mL). Demonstrated strong long-term immunity; no urgent need for routine booster.

Van Mulder et al., 2019 [74]	Belgium	Randomized clinical trial	Healthy adults aged 18– 35 years, fully vaccinated against hepatitis B at least five years before enrollment	48 participants	HBVac via VAX-ID™ device	Compared immunogenicity and safety of intradermal (ID) vs. intramuscular (IM) hepatitis B booster using a novel device (VAX-ID™)	All participants showed anamnestic response 14 days post-booster; ID groups had 3-fold higher immune response at days 14 and 30 compared to IM group. Titers remained elevated through day 210. Local adverse reactions were more frequent with ID route. VAX-ID™ provided effective, standardized ID delivery.
Van Damme et al., 2019 [75]	Belgium	Randomized clinical trial	Adults aged 40–60 years previously vaccinated with 3–4 doses of recombinant hepatitis B vaccine 20–30 years earlier	101 participants	HBVac	Evaluated persistence of anti-HBs antibodies and immune memory 20–30 years after adult vaccination without booster	90.1% had HBsAb ≥10 mIU/mL before challenge dose; 84.2% and 100% showed anamnestic response at 7 and 30 days post-challenge. Memory B and CD4+ T cell responses increased markedly after challenge, con- firming durable immune memory and long-term protection without need for booster in immunocompetent adults.
Tfifha et al., 2019 [76]	Tunisia	Cross-sectional study	Healthy children under 6 years vaccinated per national schedule (four-dose HepB including birth dose)	240 enrolled, 180 participants	HBVac (four-do se)	Evaluated antibody persistence and anamnestic immune response after booster in young children	Seroprotection rate was 77.2%. HBsAb levels declined significantly with age. All children with low antibody levels (<10 IU/L) developed anamnestic responses after booster, confirming persistence of immune memory despite antibody decline.
Lu et al., 2019 [77]	China	Cohort study	Adults aged 15–40 years with 12-year follow-up, on 0-,1-,6-month or 0-,6-month schedule	341 (3-dose) and 288 (2-dose)	HBVac	Compared long-term HBsAb persistence between 2-dose and 3-dose schedules	The 3-dose schedule resulted in higher long-term seroprotection and antibody levels than the 2-dose schedule, though both schedules maintained good persistence.
Lee et al., 2019 [78]	Singapore	Cohort study	Term infants born to chronic hepatitis B carrier mothers	177 (mono-valent)	HBVac	Compared vaccine effectiveness and immunogenicity between monovalent and combination vaccine regimens in infants at risk of vertical HBV transmission	Both vaccine regimens showed similarly high effectiveness with low immunoprophylaxis failure and comparable anti-HBs levels at 9 months.
Giao et al., 2019 [79]	Vietnam	Cross-sectional study	Parents of children aged 12–24 months, between February 2016 and July 2017	768 participants	HBVac (EPI birth dose + 3-dose series)	Assessed parental attitudes and practices regarding HepB vaccination and timeliness	Only 34% of children completed all four doses on time, and 45.2% received the birth dose within 24 hours; parental awareness, rural residence, and advice from health workers improved vaccination practices.
Zhou et al., 2018 [80]	China	Randomized clinical trial	Chronic hepatitis B patients (44 HBeAg seroconverted patients and 49 randomly picked non-seroconverted patients)	93 participants	HBsAg-HBIG therapeutic vaccine (YIC)	Evaluated cytokine/chemokine biomarkers to distinguish HBeAg seroconverted from non-converted patients and predict therapeutic vaccine response	Individual cytokines did not strongly predict response. Combined analysis of 14 cytokines/chemokines (notably IL-10, IL-33, MIP-1α) correlated best with HBeAg seroconversion, showing 0.59 sensitivity and 0.8 specificity. Multi-marker analysis may help select appropriate patients for YIC therapy.
Zhang et al., 2018 [81]	China	Cohort study	Adults aged 18–59 years, 3-year follow-up	6250 enrolled, 4701 participated in the follow-up	HBVac	Assessed risk factors for hepatitis B breakthrough infection (HBBI) after vaccination, including antibody response	HBBI rate was 5.36%; highest in 18–29 years (7.33%). Breakthrough infection varied by township and anti-HBs response. Hyporesponse to HepB was independently associated with HBBI. No participants were HBsAg positive. Suggests monitoring young adults for new HBV infection.

Wu et al., 2018 [82]	China	Cross-sectional study	Children aged 5–15 years after primary HepB immunization between 2009 to 2010	559 children	HBVac (booster)	Evaluated antibody response and seroprotection after booster doses in children with varying pre-booster anti-HBs levels	Booster vaccination provided high seroprotection: Group I (<10 mIU/mL) achieved 91.05% at 5 years; Group II (≥10 mIU/mL) maintained 98.21%. 3-dose boosters recommended for children who lost immunity; single booster sufficient for those with protective levels. Younger children (5–9 y) had higher GMT initially.
Weinberger et al., 2018 [83]	Germany	Observational study	Healthy young (20–40 y) and elderly (>60 y) adults	41 (24 young, 17 elderly)	HBVac	Compared primary versus booster antibody responses and gene expression profiles in young vs. older adults	Elderly had impaired and delayed responses to primary vaccination; non-responders only in elderly. Booster responses were comparable between age groups. Gene expression profiling revealed age- and response-related signatures during primary vaccination but not during booster. Age affects primary more than booster vaccine responses.
Wei et al., 2018 [84]	China	Cohort study	Infants born to HBsAg-positive mothers	1,177 infants (545 in 100 IU HBIG group, 632 in 200 IU HBIG group)	HBVac + HBIG	Compared efficacy of two HBIG dosages combined with HepB to prevent MTCT of HBV	MTCT rates were low and similar between groups at 7 months (1.5% vs 1.9%) and 12 months. Anti-HBs positivity rates were >97% in both groups. 100 IU HBIG is sufficient when combined with HepB series.
Wang et al., 2018 [85]	China	Cross-sectional study	Healthy young adults aged 18–25 years, 2-year follow-up	353 participants	HBVac (20 µg 3-dose, 60 µg 2-dose)	Compared immunogenicity of different dosages and schedules 2 years after primary vaccination	20 µg 3-dose: 98.3% seroprotection, GMC 427.5 mIU/mL vs 60 µg 2-dose: 85–88%, 89–90 mIU/mL. Standard 20 µg 0-1-6 mo schedule recommended for adults
Saffar et al., 2018 [86]	Iran	Observational study	Young adults with an age range from 18.8–20.5 years, Youths with neo- natal HB vaccination	252 participants	HBVac (single neonatal dose)	Duration of protection from HB vaccination; immune response to booster dose	131 participants were serosusceptible (<10 IU/L); 114 responded to booster; ~90% preserved long-term protection; study does not support routine HB booster
Pronocitro et al., 2018 [87]	Indonesia	Cross-sectional study	Children aged 8 months to 5 years who completed HB vaccination	52 Children	HBVac	Protective HBsAb levels and correlation with CD4 T cell counts	Median anti-HBs titer 72.965 IU/L; 73.1% had protective antibody levels; mean CD4 count 49.73% ± 29.75; no correlation between anti-HBs titer and CD4 count; weak negative correlation between age and anti-HBs titer
Kishino et al., 2018 [88]	Japan	Randomized clinical trial	Healthy young adults aged 20–35 years, multi- center trial from December 2011 through November 2012.	722 participants	HBVac	Immunogenicity, safety, and tolerability of a modified-process HB vaccine compared to licensed Heptavax®-II	mpHBV SC seroprotection non-inferior to Heptavax®-II SC; higher anti-HBs GMTs. AEs mostly mild-moderate; no serious vaccine-related AEs. IM administration more immunogenic and well-tolerated
Jackson et al., 2018 [89]	USA	Randomized clinical trial	Adults aged 18–70 years, including participants with type 2 diabetes	8374 randomized (6826 per-protocol)	HBVac	Immunogenicity of a two-dose investigational HB vaccine compared to licensed three-dose vaccine; effect in adults with factors reducing vaccine response	Seroprotection rate at week 28 in diabetic participants: 90.0% (HBsAg-1018) vs 65.1% (HBsAg-Eng); in overall population, HBsAg-1018 had significantly higher seroprotection than HBsAg-Eng; fewer doses and shorter schedule with greater immunogenicity
Hyer et al., 2018 [90]	USA	Randomized clinical trial	Adults ≥18 years	Not specified (pooled from 3 trials)	HBVac	Safety of a two-dose investigational HB vaccine	Similar safety profile to HBsAg-Eng; post-injection reactions, AEs, and serious AEs balanced; transient anti-beta2 glycoprotein 1 IgM increase not associated with thrombosis; improved immunogenicity and fewer doses over shorter schedule

Zhu et al., 2017 [91]	China	Randomized clinical trial	Healthy neonates (born to HBV-positive or HBV-negative mothers)	1740 participants (894 Hepavax-Gene TF, 734 Engerix-B)	HBVac	Comparative efficacy, safety, and immunogenicity of Hepavax-Gene TF and Engerix-B in preventing mother-to-child HBV transmission	>95% prevention of HBV transmission; seroprotection rates >90% at 1 and 6 months; Hepavax-Gene TF noninferior to Engerix-B in efficacy and immunogenicity; both vaccines well tolerated with similar safety profiles
Wang et al., 2017 [92]	China	Cohort study	Infants born to HBsAg(+) mothers, From January 2010 to January 2014	328 mother–infant pairs	HBVac	Association between maternal HBsAg and infant immune response to HB vaccination	6.7% of infants showed non-response (HBsAb <10 mIU/mL); non-response higher in HBsAg(+) newborns (13%) vs HBsAg(–) (5%); high maternal HBsAg titers linked to infant HBsAg positivity at birth; HBsAg transplacental transfer demonstrated; poor vaccine response associated with maternal HBsAg transfer
Verso et al., 2017 [93]	Italy	Cross-sectional study	Undergraduate and postgraduate healthcare students vaccinated in infancy or adolescence, from January 2014- July 2016	2,114 participants	HBVac	Long-term immunogenicity and predictors of anti-HBs titers two decades post-vaccination	38.1% had HBsAb <10 IU/L (non-protective); lower titers associated with younger age, nursing/midwifery program, and infant vaccination; recommends assessing HBV markers in healthcare trainees and providing booster doses for low responders
Tan et al., 2017 [94]	Singapore	Cohort study	Preterm infants (<37 weeks gestation) born to HBsAg-positive mothers, from June 2009 to December 2015	24 mother–infant pairs (23 survivors)	HBVac (3- or 4-dose schedule)	Serologic response to hepatitis B vaccination in pre- term infants of HBsAg-positive mothers	95.5% achieved protective HBsAb (>10 IU/L); all infants with birth weight <2 kg achieved seroprotective levels; no HBsAg positivity in any surviving infant; ACIP schedule effective for this high-risk group
Tai et al., 2017 [95]	Taiwan	Cohort study	HBeAg-seropositive children with chronic HBV infection (vaccine failure vs nonvaccinated)	356 (105 vaccine failure; 251 nonvaccinated)	HBVac (3-dose infant series)	Long-term natural course of chronic HBV infection in vaccine failure vs nonvaccinated children	Vaccine failure group had significantly lower cumulative HBeAg sero-conversion (30.5% vs 77.7%); higher frequency of HBV genotype C infection and maternal HBsAg positivity; vaccine failure, genotype C, and maternal HBsAg positivity linked to delayed seroconversion
Pinto et al., 2017 [96]	Canada	Prospective study	Adolescents aged 10–11 and 15–16 years vaccinated in infancy (2-, 4-, 6-month schedule)	359 (140 young, 219 old)	HBVac (infant 3-dose series)	Assessment of residual immunity and immune memory 10–16 years after infant HBV vaccination	78% (younger) and 64% (older) had HBsAb <12 mIU/mL at baseline; after booster, ≥91% developed protective titers; strong anamnestic response confirmed immune memory persistence; weaker responses in older adolescents; partial memory loss observed in small subset
Lu et al., 2017 [97]	China	Observational study	Neonates born to HBsAg(+)/HBeAg(–) mothers	884 (132 vaccine alone; 752 vaccine + HBIG)	HBVac (with or without HBIG)	Efficacy of HBV vaccine alone vs vaccine + HBIG for preventing perinatal HBV transmission	HBV transmission: 0.1% (with HBIG) vs 0.0% (vaccine alone); anti-HBs response rates >97% in both groups; vaccine alone produced higher anti-HBs GMC at 7 and 12 months; no horizontal HBV transmission; vaccine alone sufficient for HBsAg(+)/HBeAg(–) mothers
Lu et al., 2017 [98]	China	Prospective interventional study	Children aged 5–15 years with negative anti-HBs (<10 mIU/mL) after primary HBV immunization,	759 enrolled	HBVac (3-dose booster, 20 μg, 0–1–6-month)	Evaluation of antibody re-sponse after booster vaccination in previously immunized children	Seroprotective rates: 93.8% (1 mo post–first dose), 100% (1 mo post–third), 83.6% (1 yr), 73.4% (5 yrs); stronger response in children with pre-booster titers ≥1.0 mIU/mL; booster effect correlated with pre-booster titer level; robust long-term response achievable with boosters
Lee et al., 2017 [99]	South Korea	Cross-sectional study	Children aged 7 months– 17 years who underwent HBV antigen/antibody testing, from March 2012 to April 2015	5,655 children	HBVac	Evaluation of changes in HBsAb titers over time and response to booster in Korean children	Continuous decline in HBsAb titers with age; 50% had titers <10 IU/L after 7 years; lowest titers observed by age 14; after a single booster, 96% seroconverted (HBsAb ≥10 IU/L); confirms waning immunity but strong booster response

Huynh et al., 2017 [100]	Canada	Cross-sectional study	General population of Nunavut (aged 1 week– 93 years), from April 2013 to April 2014	4,771 serum specimens	HBVac	Evaluated long-term impact of 20 years of universal infant HBV vaccination on HBV prevalence and immune protection	HBV prevalence ↓ from >2% to 1.2%. Post-1980 birth: 10-fold lower exposure (1.8% vs 19.8%). HBsAg+ mainly pre-1980 (2.5%), rare in vaccinated (0.3%). Vaccine antibodies waned <10% by 5–19 y. Vaccination reduced infection but waning immunity/residual transmission remain.
Wang et al., 2016 [101]	China	Randomized clinical trial	Healthy adults aged 25– 55 years, HBsAg-, anti-HBs-, and anti-HBc-negative	1,169 participants	HBVac	Compared immunogenicity and safety of high-dose rapid 2-dose vs. standard 3-dose hepatitis B vaccines	60 mg HBVac has a satisfactory safety and seroprotection rate in adult, it could be used in rapid adult hepatitis B immunization; BMI, age > 40, and smoking reduced responses
Van Der Meeren et al., 2016 [102]	Germany	Randomized clinical trial	Adolescents aged 15–16 years vaccinated in in- fancy (3-dose series, last dose <18 months), 10 centers between July 2013 and February 2014	293 participants	HBVac (10 µg HBsAg)	Evaluated long-term immunity and immune memory 15–16 years after infant HBV vaccination	Pre-challenge seroprotection: 65.4%; post-challenge: 97.9%; 96.9% showed an anamnestic response; GMC increased 150-fold post-challenge (from ~27.6 to 4134.9 mIU/mL); booster well tolerated, confirming durable immune memory without need for routine booster
Klushkina et al., 2016 [103]	Russia	Cross-sectional study	General population across six regions, including children ≤5 years, women of childbearing age, and adults ≥30 years	6,217 sera samples	HBVac	Evaluated HBV prevalence, infection-associated morbidity/mortality, and circulation of immune escape variants 10 years post-vaccination	HBsAg prevalence: 1.2–8.2%; anti-HBc: 13.0–46.2%; children ≤5 yrs: 0– 2.4%; women of childbearing age: 0.6–10.5%; adults ≥30 yrs: 1.9–8.1%. Birth-dose coverage (96.1–99.6%) increased effectiveness 10–21× vs. lower coverage. HBV DNA detected in 63 samples; immune escape variants rare (3% within a-determinant). Universal vaccination caused a sharp decline in acute HB while maintaining vaccine efficacy with low variant circulation.
Katoon-izadeh et al., 2016 [104]	Iran	Cohort study	Healthy children/adolescents aged 10–18 years born to HBV-infected parents and vaccinated in infancy (3-dose neonatal HBV vaccine series)	541 participants	HBVac	Evaluated persistence of immunity and anamnestic response 10–18 years post-vaccination	Seroprotection (anti-HBs ≥10 IU/mL): 48% in 10–11 years vs. 26.5% in 15– 18 years (p=0.008); anamnestic response: 96% in youngest vs. 75% in oldest (p=0.005); waning immunity with age; predictors of response to booster: age (OR=0.80, p=0.01) and pre-booster HBsAb (OR=0.37, p=0.01); suggests declining immune memory over time.
Hsu et al., 2015 [105]	Taiwan	Population-based study	Children and adolescents (<18 years) from six national surveys (1984– 2009); cohorts before and after universal infant HBV vaccination	1,328 participants	HBVac	Evaluated impact of universal infant immunization on occult HBV infection (OBI) prevalence and mutation profile	OBI frequency ↓ 160.7→11.5/10^4 HBsAg– subjects. 0% in vaccinated anti-HBc– vs 1.8% unvaccinated (p=0.007); higher OBI in vaccinated anti-HBc+ (4.8%) vs unvaccinated (1.7%). Cases had low viral load, more 'a' determinant mutation. Universal infant vaccination reduced overt and occult HBV
Qu et al., 2014 [106]	China	Randomized con- trolled trial	Newborns in Qidong, China	72,807 total (38,366 vaccinated, 34,441 controls)	HBVac (3-dose neonatal series)	Long-term efficacy of neo- natal HBV vaccination on HBsAg seroprevalence, primary liver cancer (PLC), and other liver diseases over 30 years	Neonatal vaccination: PLC ↓84% (95% CI 23–97%), severe liver mortality ↓70% (15–89%), infant fulminant hepatitis ↓69% (34–85%). Catch-up at 10–14 years less effective (21% vs 72%). Booster at 10–14 years ↓ HBsAg in children of HBsAg+ mothers (HR 0.68). Emphasizes neonatal vaccination and targeted adolescent booster

Pan et al., 2014 [107]	China	Randomized clinical trial	Healthy adults (16–60 years) who were non-responders (anti-HBs <10 mIU/mL) after routine HB vaccination	1,091 participants	HBVac with high antigen content	Evaluated immunogenicity and safety of high-dose booster HB vaccines in adults who failed to respond to standard regimen	Seroconversion ≥92.1% (60 μg), ≥87.1% (30 μg), ≥83.0% (10 μg) after second dose; GMCs ≥286, ≥164, ≥110 mIU/mL respectively; all formulations well tolerated, with mild/moderate local/systemic reactions (32.3% overall); no serious AEs; high-dose vaccine effective and safe in non-responders

AEs – Adverse event(s); AR – Attack rate; CHO – Chinese hamster ovary (cell line used in vaccine production); EPI – Expanded programmed on immunization; GMC – Geometric mean concentration; GMT – Geometric mean titer; HBV – Hepatitis B virus; HBsAg – Hepatitis B surface antigen; HBeAg – Hepatitis B e antigen; HBIG – Hepatitis B immunoglobulin; HBBI – Hepatitis B breakthrough infection; HBVac – Hepatitis B vaccine; MTCT – Mother-to-child transmission; OBI – Occult hepatitis B virus infection; PVST – Post-vaccination serologic testing; POCT – Point-of-care testing; PY – Person-years; TDF – Tenofovir disoproxil fumarate; RRR – Relative risk reduction; VBI – Vaccine breakthrough infection.

Table S6. Summary of hepatitis B vaccination studies and therapeutic hepatitis C vaccine candidates in individuals with chronic HCV infection, published between 2000 and 2025

Author/Year	Country	Type of Study	Population	Sample Size	Type of Vaccine	Epidemiological Characteristics / Study Focus	Main Findings / Results
Yan et al., 2023 [108]	China	Randomized controlled trial	Adults' patients with Chronic HCV and matched healthy controls	300 participants (100 CHC, 200 Control)	HBVac (3-dose schedule + booster for non-protective titers)	Evaluation of long-term persistence of humoral and cell-mediated immune responses to HBV vaccination among CHC patients compared to healthy controls	At 5 years post-vaccination, only 56.6% of CHC patients retained protective anti-HBs titers (≥10 mIU/mL) vs. 70.8% of controls ($p < 0.05$). GMC of anti-HBs was lower in CHC group (16.95 vs. 37.34 mIU/mL, $p<0.05$). Booster dose restored GMC and anamnestic responses in both groups (no difference post-booster). HCV infection independently predicted lower anti-HBs levels. Elevated HBsAg-specific IL-6 responses in CHC patients correlated with impaired long-term humoral immunity. Spot-forming cells (SFCs) a correlated with anti-HBs levels.
Medeiros et al., 2023 [109]	Brazil	Randomized controlled trial	Adults with un-treated chronic HCV (non-cirrhotic) and healthy HCV-negative controls	141 enrolled, 128 followed up	HBVac (20 µg or 40 µg at 0, 1, 6 months)	Evaluation of efficacy of double versus standard HBV vaccine dose in eliciting protective anti-HBs response among non-cirrhotic chronic HCV patients	Seroprotection rate: 76.7% (40 µg) vs 73.5% (20 µg) in HCV-positive groups ($p = 0.68$); 91.2% in controls ($p=0.011$ and 0.003). Double-dose regimen did not improve vaccine response (OR = 0.63, $p = 0.33$). Fourth booster dose induced seroconversion in 45.5% (40 µg) vs 21.4% (20 µg) non-responders. HCV infection impairs HBV vaccine response; double dose does not overcome hypo responsiveness, though a fourth dose may improve seroconversion.
Abd El-Wahab et al., 2021 [110]	Egypt	Observational study	Chronic HCV adults' patients after SVR with direct-acting antivirals (DAAs), including sub- group with isolated anti-HBc positivity	118 enrolled (98 vaccinated)	HBVac (standard 3-dose regimen)	Evaluation of HBV vaccine effectiveness and predictors of hyporesponsiveness among DAA-treated HCV patients, with focus on isolated anti-HBc status	Overall vaccine response rate 57.1%, but only 5.1% achieved seroprotective anti-HBs levels. Response was significantly lower among patients with isolated anti-HBc (5.6%) compared to those without (64.5%). Multivariate analysis: advanced age (OR=1.09; 95% CI 1.02–1.17) and isolated anti-HBc (OR = 39.59; 95% CI 7.98–196.63) predicted vaccine non-response. Hyporesponsiveness persists despite SVR after DAA therapy.
Colombatto et al., 2014 [111]	Italy	Randomized controlled trial	Chronic HCV adults patients undergoing retreatment with Peg-IFNα2a + ribavirin, from January 2005 to June 2008	78 patients (Vaccine = 23; P/R = 25; P/R + V= 30)	HCVac (100 µg/0.5 mL IM; weeks 0, 4, 8, 12, 24, 28, 32, 36)	Evaluation of safety, immunogenicity, and impact on viral kinetics when E1E2-MF59 vaccine was combined with Peg-IFNα2a + ribavirin in previously treated chronic HCV patients	The vaccine was safe and immunogenic. Sustained virologic response (SVR) achieved in 4 patients (P/R + V) vs 2 patients (P/R). Combination group (P/R + V) showed higher NOB anti- body titers and stronger E1E2-specific $CD4^+$ T-cell proliferation at weeks 12–16 ($p < 0.05$). Among patients with strong early IFN antiviral effect ($\varepsilon \geq 0.8$), P/R + V group had significantly lower HCV-RNA levels at week 16 ($p = 0.026$). HCVac enhanced cellular and humoral immune responses and improved viral clearance dynamics when combined with standard antiviral therapy.

Weiland et al., 2013 [112]	Sweden	Randomized controlled trial	Treatment-naïve adults' patients with chronic HCV genotype 1 infection	12 patients (8 received sub-sequent standard-of-care therapy)	HCVac (167 µg – 1,500 µg; 4 doses, monthly)	Evaluation of safety, immunogenicity, and antiviral effects of a therapeutic DNA vaccine against HCV, followed by standard peg-IFN and ribavirin therapy	Vaccine was safe and well tolerated. Induced significant increases in IFN-γ–producing NS3-specific T-cell responses within first 6 weeks. Five patients showed 0.6–2.4 $\log_{10}$ reduction in HCV RNA during vaccination. Among 8 patients receiving SOC therapy after vaccination, 6 achieved SVR. Demonstrated transient antiviral effects and potential for combination with standard therapy in chronic HCV genotype 1 infection.

Anti-HBc – Antibody against hepatitis B core antigen; Anti-HBs – Antibody against hepatitis B surface antigen; CHC – Chronic hepatitis C; CI – Confidence interval; DAAs – Direct-acting antivirals; GMC – Geometric mean concentration; HBVac – Hepatitis B vaccine; HCV – Hepatitis C virus; HCVac – Hepatitis C vaccine; IM – Intramuscular; NOB – Neutralizing-of-binding antibodies; OR – Odds ratio; P/R – Peg-IFNα2a + Ribavirin; P/R + V – Combination of Peg-IFNα2a + Ribavirin with vaccine; Peg-IFNα2a – Pegylated interferon alpha-2a; SVR – Sustained virologic response.

Table S7. Summary of hepatitis B vaccination studies and hepatitis E vaccine studies in individuals exposed to or infected with HEV, published between 2000 and 2025

Author/Year	Country	Type of Study	Population	Sample Size	Type of Vaccine	Epidemiological Characteristics / Study Focus	Main Findings / Results
Grzegorzewska et al., 2019 [113]	Poland	Cross-sectional study	Hemodialysis patients (HBV/HCV negative) with or without prior HEV exposure	76 patients (31 HEV IgG positive, 45 HEV negative)	HBVac (3-dose series with boosters as needed)	Assessment of HBV vaccine response and correlation with circulating IFN-λ3 levels in patients, comparing HEV-exposed vs non-exposed individuals	No difference in anti-HBs titers or IFN-λ3 levels between HEV-exposed and non-exposed HD patients. In vaccine responders, plasma IFN-λ3 positively correlated with anti-HBs titers ($r = 0.505$, $p = 0.01$ for HEV-exposed; $r = 0.523$, $p = 0.001$ for controls). Past HEV infection did not impair HBV vaccine response.
Chen et al., 2019 [114]	China	Randomized controlled study	Healthy adults aged ≥18 years	126 participants (63 accelerated, 63 routine schedule)	HBVac (3-dose schedule; accelerated: 0, 7, 21 days; routine: 0, 1, 6 months)	Evaluation of immunogenicity and safety of accelerated HEV vaccination schedule versus routine schedule	Seropositive rates were 100% in both groups (63 accelerated, 63 routine schedule); GMCs were 8.51 WHO U/mL (accelerated) vs 9.67 WHO U/mL (routine). Accelerated schedule was non-inferior to routine. Solicited AEs: 32.3% (accelerated) vs 30.2% (routine), mostly moderate. Accelerated schedule provides rapid protective immunity and is safe, suitable for short-notice travelers or outbreak situations.
Su et al., 2017 [115]	China	Randomized controlled study	Baseline seropositive adults from a HEV vaccine trial	4273 participants (2242 placebo, 2031 vaccine recipients)	HEVac	Analysis of persistence of naturally acquired anti-HEV IgG and long-term effect of vaccination on antibody durability	Naturally acquired anti-HEV IgG declined steadily, with 50% of placebo recipients projected to lose detectable antibodies by 14.5 years. HEV vaccination produced substantially more durable responses: 82.1–99.4% of vaccines were predicted to remain seropositive at 30 years. These projections were based on a nonlinear decay model, although the original study did not provide confidence intervals or detailed modeling parameters. Vaccination therefore offers robust and sustained long-term immunity compared with natural infection.
Cao et al., 2017 [116]	China	Randomized controlled study	Healthy adults aged 16–65 years	120 participants	Candidate HEV vaccine	Evaluation of safety and tolerability of novel HEV vaccine at different dosages	Vaccine was safe and well tolerated at all doses. The incidence of solicited local ARs within 7 days in the subjects receiving the 20 μg, 30 μg, 40 μg dose and the control vaccine were 6.67%, 26.67%, 30.00% and 40.00%, respectively. Solicited local adverse reactions were lower in experimental groups than control ($p = 0.027$); systemic ARs and lab test changes were mild or moderate with no significant differences. No vaccine-related serious AEs occurred. The 30 μg dose was selected for further efficacy studies.

Zhang et al., 2015 [117]	China	Randomized controlled study	Healthy adults aged 16–65 years	60 cases (7 in the HEV vaccine group and 53 cases in the control group)	HEVac, 3-dose schedule at 0, 1, 6 months; HBV vaccine as control	Evaluation of long-term efficacy, immunogenicity, and safety of HEV vaccine over 4.5 years	Vaccine efficacy was 86.8% (95% CI: 71–94). 60 cases of hepatitis E were identified; 7 cases were confirmed in the vaccine group (0.3 cases per 10,000 person-years), and 53 cases in the control group (2.1 cases per 10,000 person-years), representing a vaccine efficacy of 86.8% (95% confidence interval, 71 to 94) in the modified intention- to-treat analysis. Of seronegative participants, 87% maintained anti-HEV antibodies for ≥4.5 years. AE rates were similar between vaccine and control groups. HEV vaccination induced long-lasting immunity and protection.

AE(s) – Adverse event(s), anti-HBs – Antibody to hepatitis B surface antigen; HBV – Hepatitis B virus; HBVac – Hepatitis B vaccine, HCV – Hepatitis C virus; HD – Hemodialysis, HEV – Hepatitis E virus; IgG – Immunoglobulin G; IFN-λ3 – Interferon lambda 3, GMC – Geometric mean concentration.

Table S8. Summary of articles reporting characteristics of hepatitis vaccines in human's non-viral hepatology disease, published between 2000 and 2025

Author/Year	Country	Type of Study	Population	Sample Size	Type of Vaccine	Epidemiological Characteristics / Study Focus	Main Findings / Results
Horta et al., 2022 [118]	Spain	Observational study	Patients with CLD (non-cirrhotic and cirrhotic) who were seronegative for HBV	125 participants	HBVac (4-dose or 3-dose schedule)	Assessment of seroconversion, kinetics of anti-HBs titers, and factors influencing response to adjuvanted HBV vaccination in chronic liver disease	Anti-HBs was measured at 2, 6, and 12 months post-vaccination. A responder was primarily defined as an Anti-HBs level >1,000 IU/L, indicating a robust serological response. An additional higher threshold (>100,000 IU/L) was included to describe very high antibody responses and to further characterize the magnitude of immunogenicity. Overall response at 2 months was 76.8% (FENDRIX® 83.7%, BVAXPRO® 72.4%). GMTs were similar, though FENDRIX® tended to be higher, suggesting slightly better efficacy. Response rates declined over time: 72.8% at 6 months and 59.7% at 12 months.
Amjad et al., 2021 [119]	USA	Cohort study	Adults with CLD, including cirrhotic	166 participants (60 Heplisav-B; 106 Engerix-B)	HBVac	Comparison of immunogenicity (seroconversion) between two HBV vaccine regimens in CLD patients and identification of predictors of vaccine response	Seroprotective HBsAb (>10 mIU/mL) achieved in 63% with Heplisav-B vs 45% with Engerix-B (p=0.03). Cirrhosis, COPD, and renal failure reduced likelihood of achieving immunity. Heplisav-B re- cipients had 2.7-fold higher odds of seroconversion (aOR 2.74; 95% CI 1.31–5.71). Heplisav-B is more immunogenic in CLD patients.
Wigg et al., 2019 [120]	Australia	Prospective comparative study	Adults with cirrhosis	HAV: 73, HBV: 97	HAVac and HBVac	Evaluation of immunogen- icity of accelerated high-dose vs standard-dose vaccination schedules in cirrhotic patients	A potential benefit of an initial HDA HAV regimen in all cirrhotic patients. Routine use of the initial HDA HBV regimen is not sup- ported, but HDA boosting in initial non-responders showed a clinically significant 23% improvement in response rate.
Duan et al., 2019 [121]	China	Observational study	LT recipients with HBV-related liver disease	78 responders	HBVac (post-transplant)	Evaluation of long-term outcomes and safety after discontinuation of HBIG and/or nucleotide analogues (NAs) in vaccine responders	All patients discontinued HBIG; 36 discontinued both HBIG and NAs. Four patients experienced HBV reinfection associated with escape mutations. No death or graft loss occurred. Careful withdrawal of HBIG and/or NAs is feasible and safe in HBV-vaccine responder's post-transplant.
Wang et al., 2017 [122]	China	Cohort study	Adult LDLT anti-HBc-positive grafts, anti-HBs-negative pre-transplant	71 patients	HBVac (pre- and post-transplant)	Evaluation of efficacy of HBV vaccination in preventing DNHB after LDLT and ability to discontinue antiviral prophylaxis	Cohort stratified into three groups: Group 1 (pre-transplant an- ti-HBs >1000 IU/L, n=24), Group 2 (pre-transplant <1000 IU/L, post-transplant lamivudine, n=30), Group 3 (low titer non-responders, n=17). All DNHB occurred in Group 3 (17.6% incidence). Maintaining post-transplant anti-HBs >100 IU/L prevented DNHB. Pre-transplant anti-HBs >1000 IU/L predicted sustained post-transplant protective titers. Active vaccination allowed safe discontinuation of antivirals in responders.
Seniuta et al., 2017 [123]	Poland	Observational study	Children with NAFLD and healthy controls	54 NAFLD, 120 controls	HBVac (3-dose schedule + booster for non-immune)	Assessment of immune response to primary HBV vaccination and booster dose in children with NAFLD compared to healthy children	32% of NAFLD patients had anti-HBs <10 IU/l vs 19% in controls (p=0.07). All NAFLD children responded to booster (GMC 239 IU/l, median 313 IU/l, range 19–1000), while 10/13 controls responded (GMC 148 IU/l, median 686 IU/l, range 1–1000). Suggests NAFLD children respond similarly to healthy children after booster.

Lu et al., 2014 [124]	China	Cohort study	OLT recipients for HBV-related end-stage liver disease	200 OLT recipients	HBVac (4-dose schedule at 0, 1, 2, and 6 months)	Evaluate reestablishment of active immunity against HBV graft reinfection under low-dose HBIG + antiviral prophylaxis	Active immunity achieved in 50/200 (25%). 24 patients discontinued HBIG without reinfection (follow-up 26.1 ± 7.05 months). 21 discontinued both HBIG and antivirals (follow-up 39.9 ± 15.5 months), 4 became HBsAg positive. No deaths or graft loss due to HBV reinfection. Vaccination feasible; HBIG withdrawal reasonable, but discontinuing antivirals requires caution.
Bagheri Lankarani et al., 2014 [125]	Iran	Randomized controlled trial	Patients with liver cirrhosis awaiting LT	56 (28 vaccine + G-CSF, 28 vaccine + placebo)	Double-dose HBVac (40 μg at weeks 0, 4, 8) ± G-CSF	Evaluated the adjuvant role of G-CSF in enhancing immunogenicity of HBV vaccine in cirrhotic patients	No significant difference in antibody titers between G-CSF and placebo groups after each vaccination ($p > 0.05$). G-CSF group showed a faster rise in antibody titers, though not significantly higher overall. Concluded G-CSF not superior to placebo for achieving protective titers but may accelerate response.
Takaki et al., 2013 [126]	Japan	Observational study	Adult LT recipients (HBV-related liver cirrhosis or acute HBV liver failure)	27 transplants (5 ALF-OLT, 22 LC-OLT)	HBVac	Evaluated immune response to HBV vaccination post-living donor liver transplantation and factors influencing response	All 5 ALF-OLT patients were good responders after median 4 vaccinations; 9/22 LC-OLT patients responded after median 19 vaccinations. Donor factors (higher age, marital status, high anti-HBs titer) predicted better response. Good responders showed strong IFN-γ responses. Vaccination effective in select recipients; donor immune status may enhance recipient vaccine response.
Ferreira et al., 2003 [127]	Brazil	Randomized controlled trial	Children (1–16 years) with CLD vs. healthy controls	89 patients (34 with CLD, 55 controls)	HAVac	Evaluated immunogenicity and safety of HAV vaccine in pediatric chronic liver disease	Seroconversion at 4 weeks: 76% (CLD) vs 94% (controls); after 2nd dose: 97% vs 100%. GMTs were lower in CLD group (812 vs 2345 mIU/mL) but protective. Vaccine was safe and well-tolerated. HAV vaccine immunogenic even in CLD, though with lower antibody titers.
Chang et al., 2003 [128]	South Korea	Observational study	Pediatric LT recipients (anti-HBc$^+$ and anti-HBc$^-$ donors)	19 trans-plants	HBVac	Evaluated efficacy of HBV vaccination post-liver transplantation for pre-venting de novo HBV infection, especially from anti-HBc$^+$ grafts	17/19 (89.5%) responded to vaccination. Among anti-HBc$^+$ donor group: 3 high responders (HBsAb ≥1000 IU/L), 4 low responders, 2 non-responders (1 developed de novo HBV). Among anti-HBc$^-$ donors: 5 high and 5 low responders. HBV vaccination showed partial protection, especially in pediatric recipients of anti-HBc$^+$ grafts.
Bienzle et al., 2003 [129]	Germany	Randomized controlled trial	LT recipients for HBV-related dis-eases under continuous HBIG	20 trans-plants	HBVac	Evaluated immunogenicity and safety of novel adjuvanted HBV vaccines (two formulations) in HBV-related liver transplant recipients on HBIG prophylaxis	16/20 (80%) developed protective anti-HBs titers (Group I median: 7,293 IU/L; Group II median: 44,549 IU/L). Responders discontinued HBIG without HBV reinfection during median 13.5-month follow-up. Vaccine well tolerated; demonstrated potential to re- place long-term HBIG prophylaxis.
Smallwood et al. 2002 [130]	USA	Observational study	Patients with ESLD awaiting LT	72 patients	HAVac	Evaluated ability of ESLD patients awaiting liver transplantation to mount an immune response to HAV vaccination	Despite known impaired immunity in ESLD, many patients elicited a measurable antibody response to hepatitis A vaccination, though rates were lower than in healthy controls (97% seroconversion in healthy individuals vs. reduced rates in ESLD). Findings support ACIP recommendations to vaccinate chronic liver disease patients before transplantation.

Duca et al., 2001 [131]	Italy	Observational study	Pediatric LT recipients (>6 months post-transplant)	47 patients	HBVac (10 µg if <30 kg; 20 µg if >30 kg)	Evaluated immunogenicity and safety of HBV vaccine in pediatric liver transplant recipients who were not vaccinated pre-transplant	Overall seroconversion rate: 85% (33/47 after primary series; 7/14 after booster). Higher response with fewer immunosuppressive drugs — 100% (monotherapy), 84% (dual), 66% (triple therapy) ($p<0.01$). Vaccine was safe and well-tolerated, with only mild local pain (2.6%). Demonstrated acceptable immune response even in immunosuppressed post-transplant children.
Arslan et al., 2001 [132]	Turkey	Cohort study	Adult OLT with ESLD	356 trans-plants	HBVac (double-dose: 0, 2 weeks, 4 weeks, 6 months)	Assessed efficacy of double-dose, accelerated HBV vaccination pre- and post-OLT and factors affecting seroconversion and antibody persistence	Seroconversion pre-OLT: 36% (128/356); post-OLT 1 year: 11.6% (41/353); 2 years: 8% (26/325). GMC: pre-OLT 86.7 IU/L → 0.32 IU/L at 1 year, 0.33 IU/L at 2 years. High pre-OLT anti-HBs titers, younger age, low Child-Pugh score, HCV infection, and certain HLA types associated with better seroconversion/persistence. Rapid decline of antibodies post-transplant; overall vaccine response post-OLT is poor.
Arslan et al., 2001 [133]	Turkey	Cohort study	Adult OLT recipients, HAV seronegative	37 vaccinated; 45 controls	HAVac, 2 doses, 6 months apart	Safety and efficacy of HAV vaccination in liver trans-plant recipients; compared to unvaccinated controls and historical data from healthy and CLD patients	Seroconversion: 1 month 8% (3/37); 6 months 19% (5/26); 7 months 26% (6/23). Responders had higher WBC/lymphocyte counts and longer post-transplant duration. No seroconversion in controls. Vaccine well tolerated. Seroconversion significantly lower than healthy or pre-OLT CLD patients ($p=0.001$). Alternative strategies needed to improve response.
Arguedas et al., 2001 [134]	USA	Cohort study	Adult patients with CLD, HAV seronegative	84 patients	HAVac, 2 doses	Immunogenicity of HAV vaccination in compensated vs. decompensated liver disease; clinical predictors of seroconversion	Seroconversion after 1st dose: compensated 71.4%, decompensated 37.1% ($p<0.05$); after 2nd dose: compensated 98%, decompensated 65.7% ($p<0.05$). Median antibody titers higher in compensated vs. decompensated disease. Child-Pugh class predicted response. Advanced liver disease associated with lower seroconversion; vaccination should be done before decompensation.
Domínguez et al., 2000 [135]	Spain	Cohort study	Adult patients with ESLD awaiting LT	62 patients	HBVac, 3 doses of 40 µg at 0, 1, 2 months (with additional 3 doses in non-responders)	Immunogenicity of HBV vaccination in cirrhotic patients on LT waiting list; evaluate booster effect in non-responders	Initial response rate: 44% (22/50). Additional 3-dose schedule in non-responders raised response to 62% (31/50). Double-dose vaccination improves response in cirrhotic patients awaiting LT.

ALF – Acute liver failure; aOR – Adjusted odds ratio CLD – Chronic liver disease; CI – Confidence interval; DNHB – De novo HBV infection; ESLD – End-stage liver disease; G-CSF – Granulocyte colony-stimulating factor; GMC – Geometric mean concentration; HBIG – Hepatitis B immunoglobulin; HBV – Hepatitis B virus; HBVac – Hepatitis B vaccination; HAV – Hepatitis A virus; HDA – High-dose accelerated; IU/L – International units per liter; LDLT – Living donor liver transplant; LT – Liver transplant; NAFLD – Non-alcoholic fatty liver disease; OLT – Orthotopic liver transplant; WBC – White blood cells.

Table S9. Summary of articles reporting characteristics of hepatitis vaccines in humans with HIV, published between 2000 and 2025

Author/Year	Country	Type of Study	Population	Sample Size	Type of Vaccine	Epidemiological Characteristics / Study Focus	Main Findings / Results
Schnyder et al., 2025 [136]	Switzerland	Cohort study	PLWH, with healthy controls	75 participants (41 HIV positive, 34 control)	HAVac or com- bined HAV/Bac	Evaluated HepA vaccine immunogenicity and boost ability in immuno- compromised populations compared with healthy controls	At 8 months SCRs: adults living with HIV 97%, controls 100%; booster dose induced 71% additional seroconversion (17/24), HepA vaccine highly immunogenic in adults living with HIV and monotherapy.
Marks et al., 2025* [137]	Multinational (41 sites across 10 countries in Africa, Asia, North America, South America)	Randomized clinical trial	Adults with HIV on ART, CD4 ≥100 cells/µL, HIV RNA <1000 copies/mL, previous nonre- sponse to HepB-alum vaccine, no evidence of cur- rent or past HBV infection	561 partici- pants	HBVac -CpG vaccine (2-dose and 3-dose regimens) vs HBVac -alum vaccine (3-dose regimen)	To compare seroprotection rates of 2-dose and 3-dose HepB-CpG vs standard 3-dose HepB-alum in people with HIV who previously failed to respond to HepB-alum.	Seroprotection rates were 93.1% (2-dose HepB-CpG), 99.4% (3-dose HepB-CpG), and 80.6% (3-dose HepB-alum). Both HepB-CpG regimens were superior to HepB-alum. The 2-dose HepB-CpG was noninferior and superior to 3-dose HepB-alum (difference 12.5%, 97.5% CI 4.1–20.9%). The 3-dose HepB-CpG produced the highest antibody titers (>1000 mIU/mL in 78.1%), and >90% achieved seroprotection by week 12. No unexpected safety concerns were observed.
Fernan- dez-Fuertes et al., 2024 [138]	Spain	Cohort study	PLWH in Seville; subgroup of anti-HAV seronegative individuals	915 total participants	HAVac	Evaluation of HAV incidence, risk factors for infection, and HAV vaccination uptake and response among PLWH	HAV vaccination uptake among seronegative individuals was low (38.6%). Among those vaccinated, 20% did not respond and 1% lost immunity. Non-responders remained at risk, with 29% developing HAV infection 5–9 years later. Overall incidence was low, but outbreaks occurred mainly among non-immunized MSM, highlighting gaps in vaccination coverage and limited vaccine responsiveness in some PLWH.
Lin et al., 2023 [139]	Taiwan	Cohort study	PLWH who achieved seroconversion after hepatitis A vaccination during an acute HAV outbreak	986 patients	HAVac	Evaluated long-term durability of HAV vaccine-induced serologic responses in HIV patients on antiretroviral therapy	At 60 months post-vaccination, persistent seroprotection observed in 90.7% (ITT with LOCF) and 97.4% (PP) of participants; higher peak anti-HAV IgG titers and slower antibody decline associated with persistence. Seroreversion linked to higher BMI, lower nadir CD4 count, detectable HIV RNA at vaccination, and first dose with Havrix. Long-term protection remained high; monitoring and timely boosting recommended.
Yoshimura et al., 2022* [140]	Japan	Cohort study	Adults PLWH who were documented non-responders to standard hepatitis B vaccination	11 participants	HBVac (total dose of 10 µg)	Evaluation of immunogenicity and safety of intradermal HBV vaccination in PLWH who failed to seroconvert after standard HBV vaccination	All participants were on ART, with a mean CD4 count of 588/µL and HIV RNA <20 copies/mL in most. Anti-HBs levels rose above 10 mIU/mL in 1 participant after the first dose, 6 after two doses, and 4 after three doses, achieving seroconversion in all. Eight participants experienced mild local AEs; no serious AEs occurred. Intradermal vaccination induced protective antibody levels in all initial non-responders.

Thitipata-rakorn et al., 2022* [141]	Thailand	Cross-sectional analysis	Newly diagnosed people with HIV enrolled in same-day ART program (MSM, heterosexuals, transgender women/men)	4,011 participants	HBVac	Assessment of hepatitis B and C prevalence and associated factors among PWH in Bangkok in the context of universal HBV vaccination	Overall HBsAg and anti-HCV seroprevalence were 6.0% and 4.1%, respectively. Among MSM: HBsAg 6.2%, anti-HCV 4.7%; heterosexuals: 4.6% and 2.4%; TGW: 9.3% and 3.7%. HBsAg positivity was associated with MSM, TGW, birth before 1992, CD4 <200 cells/mm³, and ALT ≥62.5 U/L. Anti-HCV positivity was linked to MSM, age >30 years, ALT ≥62.5 U/L, creatinine clearance <60 ml/min, and syphilis. Results highlight persistent HBV infections despite universal vaccination and support routine hepatitis B and C screening, linkage to care, and catch-up vaccination for high-risk groups.
Feng et al., 2022 [142]	China	Randomized controlled trial	PLWH	338 participants	HBVac (standard-dose 20 μg or high-dose 60 μg; or 4 doses)	Evaluation of immunogenicity and safety of standard-dose versus high-dose HBV vaccination regimens among PLWH	At week 28, seroconversion rates and anti-HBs GMCs were significantly higher in both 4-dose groups (IM20×4 and IM60×4) compared with the 3-dose group (IM20×3). The GMC was numerically higher in the high-dose 4-dose group (IM60×4) than in the standard 4-dose group (IM20×4). Both 4-dose regimens significantly improved immunogenicity in PLWH.
Vargas et al., 2021 [143]	Chile	Randomized controlled trial	PLWH who previously failed HBV vaccination (an- ti-HBs <10 IU/L)	107 participants (55 standard-dose, 52 high-dose)	HBVac; standard-dose 20 μg ×3 doses vs high-dose 40 μg ×3 doses	Assessment of efficacy of high-dose vs standard-dose HBV revaccination in PLWH non-responders	High-dose vaccination led to higher serological responses than standard-dose (72% vs 51%, OR 2.48, P=0.03) and higher mean anti-HBs levels (398.0 vs 158.5 IU/L, p<0.001). Among responders, more had anti-HBs >100 IU/L in the high-dose group (80.6% vs 50%, p=0.02). At 1 year, 80% of high-dose responders maintained protective anti-HBs versus 39.1% with standard-dose (p=0.01), demonstrating superior and longer-lasting seroprotection.
Jain et al., 2021* [144]	India	Randomized controlled trial	HIV-infected children aged 18 months–12 years receiving ART	50 participants (25 per group)	HBVac (20 μg)	Comparison of seroprotection and anti-HBs titers between 3-dose and 4-dose primary immunization schedules, including an accelerated 3-dose schedule	Median anti-HBs titers at 7 months were higher in 4-dose group [225.7 IU/L] vs 3-dose [138.2 IU/L] but comparable at 12 months. Seroprotection rates were similar: 7th month (96% vs 80%), 12th month (96% vs 88%). Accelerated 3-dose schedule produced comparable titers (179.9 IU/L) and seroprotection (92%). Concluded that 3-dose double strength schedule provides comparable protection to 4-dose schedule in HIV-infected children on ART.
Feng et al., 2021 [145]	China	Randomized con- trolled trial	Adults infected with HIV	182 participants	HBVac, standard-dose 20 μg vs high-dose 60 μg (IM, 3 doses at 0, 1, 6 months)	Assessment of immunogenicity and persistence of high-dose vs stand- ard-dose HBV vaccination in HIV-infected adults	At month 7, response rate and GMC of anti-HBs were higher in the 60 μg group than 20 μg, though differences were not statistically significant. GMC declined rapidly in both groups over follow-up. Median duration of anti-HBs ≥10 mIU/mL was 20 months in 60 μg group vs 9.3 months in 20 μg group. Three-dose 60 μg regimen showed partially improved immunogenicity and persistence compared with 20 μg.

Laksana- nun et al., 2019* [146]	Thailand	Randomized controlled trial	HIV-infected adults with isolated anti-HBc antibody, stable ART, CD4 ≥200, HIV RNA <20 copies/mL	54 (27 per group)	HBVac	Compared immunogenicity and safety of 3 vs 4 standard doses of HBV vaccination in HIV-infected adults with isolated anti-HBc antibody	Anamnestic response at week 4: 25.9% (3-dose) vs 33.3% (4-dose), p=0.551. Vaccine response at week 28: 85.2% (3-dose) vs 88.9% (4-dose), p=1.000. Anti-HBs GMT at week 28: 63.8 (3-dose) vs 209.8 mIU/mL (4-dose), p=0.030. No AEs. Both regimens highly effective; vaccination recommended for all HIV-infected adults with isolated anti-HBc.
Chaiwarith et al., 2019 [147]	Thailand	Randomized controlled trial	HIV-infected adults, CD4 >200 cells/mm³, undetectable HIV RNA, negative for HBV markers	126 (42 standard doses, 43 four doses, 41 four double doses)	HBVac	Compared long-term immunogenicity (4-year follow-up) of standard 3-dose vs. 4-dose vs. 4 double-dose HBV vaccination regimens in HIV-infected adults	At ~50 months post-vaccination, responders with anti-HBs ≥10 mIU/mL: 57.1% standard, 76.7% four doses (P=0.067), 80.5% four double doses (p=0.033). Younger age and vaccination schedule associated with higher response. Four double doses regimen provided superior long-term immunity compared with standard doses.
Mancinelli et al., 2018 [148]	Malawi	Cross-sectional study	HIV-exposed infants, between 2008 and 2011	228 children	HBVac	Assessed immune response to HBV vaccination in HIV-exposed infants and correlation with HBV infection acquisition	Protective anti-HBs levels (>10 mIU/mL): 93.2% at 6 months, 87.5% at 12 months, 80.1% at 24 months. Seven children were HBsAg+ at 24 months despite anti-HBs >10 mIU/mL. HIV exposure had limited impact on vaccine response, but higher antibody levels may be required for protection.
Lin et al., 2018 [149]	Taiwan	Cross-sectional study	HIV positive adults aged at least 19 years who tested negative for anti-HAV IgG were included in this study between 2015-2016.	1,533 (1,001 vaccinated, 532 unvaccinated)	HAVac (two doses, 6 months apart)	Serologic response and effectiveness in high-risk HIV-positive adults	During follow-up, 65 patients (4.2%) acquired HAV infection: 5 (7.7%) vaccinated and 60 (92.3%) unvaccinated, yielding a vaccine effectiveness of 96.3% against acute HAV. Seroconversion at weeks 28–36 was 63.8% (intention-to-treat) and 93.7% (per-protocol). Younger age and undetectable HIV RNA were associated with higher seroconversion. Two-dose HAV vaccination was effective and protective in this at-risk population.
Kourkounti et al., 2018 [150]	Greece	Cross-sectional study	HIV-positive adults with undetectable HIV viral load from 2012 to 2016	200 participants (63 HBVaxPro, 137 Engerix)	HBVac (HBVax-Pro 40 μg vs. Engerix 20 μg)	Compared immunogenicity of a higher-dose but fewer-shot HBV schedule vs. standard 3-dose schedule in HIV-positive adults	Seroconversion rates at months 1, 12, 24: HBVaxPro 79%, 65%, 47% vs. Engerix 68%, 53%, 38%. HBVaxPro produced higher anti-HBs titers. In participants <55 years, HBVaxPro 3× more likely to provoke a response (OR=3, p=0.006). Suggests HBVax- Pro 40 μg as a more robust alternative for HIV-positive adults.
Chen et al., 2018 [151]	Taiwan	Matched case-control study	HIV-positive adults who lost protective anti-HAV antibodies after primary vaccination	225 (75 cases, 150 controls)	HAVac	Evaluated seroresponse to HAV revaccination among HIV-positive individuals whose anti- bodies waned after primary vaccination	Serological response rates higher in revaccinated patient's vs controls: week 4: 88.1% vs 10.5%, week 24: 93.3% vs 46.0%, week 28: 98.7% vs 62.7%, week 48: 98.7% vs 92.7%. Anti-HAV antibody titers significantly higher in revaccinated group. Suggests single-dose revaccination can rapidly restore seroprotection during outbreaks.

Chawansuntati et al., 2018 [152]	Thailand	Cohort study	The study included HIV-positive and HIV-negative healthy adults over 18 years who were seronegative for anti-HBs, anti-HBc, and anti-HCV, with no history of prior HBV vaccination.	172 participants (132 HIV patients, 40 healthy control)	HBVac (20 µg standard dose, 20 µg four doses, 40 µg four double doses)	Compared cytokine (TNF-α, IL-2) production and T cell responses following HBV vaccination between HIV+ and HIV- individuals	At 7 months post-vaccination, TNF-α and IL-2-producing memory CD4+ T cells were higher in healthy controls than HIV+ receiving standard doses. No differences observed in four-dose or four double-dose groups. Suggests that increasing dose/frequency may improve cell-mediated immune responses in HIV+ adults.
Siddiqui et al., 2017 [153]	India	Randomized con- trolled trial	HIV-infected unvaccinated children	55 (27 double doses, 28 standard dose)	HBVac (recombinant, 10 µg standard vs 20 µg double dose)	Compared efficacy of double vs standard dose HBV vaccination in HIV-infected children	Seroprotection (anti-HBs ≥10 mIU/mL) 12 weeks post-third dose: 60.7% standard, 74% double dose (RR 0.8, 95% CI 0.17–1.7, p=0.29). CD4 <500 associated with lower seroprotection. Double dose did not confer significant advantage over standard dose.
Bekele et al., 2017* [154]	Sweden	Interventional study	HIV-1 infected children on ART and healthy controls	112 (49 HIV-1 infected, 63 controls)	HBVac (accelerated 3-dose schedule, 4 weeks apart)	Assessed humoral and cellular responses (Tfh cells) to HBV vaccination in HIV-1 infected children	1-month post-vaccination: all except 4 HIV-infected children had protective antibody titers; titers lower in infected children (p<0.0001). Antibody titers decreased in both groups at 6 months. cTfh cell frequency and activation comparable between groups. Higher CXCL13 in infected children correlated with cTfh frequency. Suggests impaired antibody response in HIV-infected children is due to factors other than Tfh cells.
Piroth et al., 2016 [155]	France	Cross-sectional study	HIV-infected adults with isolated anti-HBc antibody, undetectable HIV RNA, between February 2011 and September 2012	54 participants	HBVac (20 µg single dose, followed by triple double-dose 40 µg if non-responsive)	Assessed immunogenicity of HBV vaccination in HIV-infected adults with isolated anti-HBc	Week 4: 46% responders. Response associated with CD4/CD8 ratio. Week 28 & month 18: 58% and 50% maintained anti-HBs ≥10 mIU/mL. Non-responders who received reinforced vaccination: 89% and 81% achieved anti-HBs ≥10 mIU/mL. Reinforced triple double-dose scheme recommended for non-responders.
Fuster et al., 2016 [156]	Spain	Cohort study	HIV-positive adults with negative HBV markers	245 participants	HBVac	Evaluated serological response to standard HBV vaccination and predictors of seroconversion in HIV-positive adults	62% seroconversion (95% CI 56–68%), mean anti-HBs 646 IU/mL. 85.5% responders had anti-HBs >100 IU/mL. Predictors of response: age <45, non-smoker, CD4/CD8 ratio >0.4. Seroconversion in this subgroup: 86%. High adherence: 97.9% completed vaccination. CD4/CD8 ratio primary predictor of positive response.
Bose et al., 2016* [157]	India	Cross-sectional study	HIV-infected children (pre-ART and on ART), from November 2010 to March 2012	33 participants	HBVac (20 µg, 4-dose double-dose schedule at 0-1-2-6 months)	Evaluated immune response to 4-dose double-dose HBV schedule in HIV-infected children	Seroconversion: 94% (31/33). Anti-HBs titres: 66% >1000 IU/L, 24% 100–1000 IU/L, 3% 10–99.99 IU/L. Only 6% did not seroconvert. High seroconversion achieved in children with mild or no immunosuppression.
Rey et al., 2015 [158]	France	Randomized controlled trial	HIV-infected adults' non-responders to prior HBV vaccination, CD4 ≥200 cells/µL	178 (90 standard-dose, 88 double-dose)	HBVac (standard 20 µg vs double 40 µg, 3 doses at 0, 4, 24 weeks)	Compared immunogenicity and safety of standard vs double-dose revaccination in HIV-infected adults who failed primary HBV vaccination	Week 28 responders: 67% standard-dose vs 74% double-dose (p=0.334). Local reactions more frequent in double-dose group (15% vs 4%). No significant difference in overall safety. Double-dose did not significantly increase response rate compared with standard-dose revaccination.

Mutwa et al., 2013* [159]	Rwanda	Cohort study	HIV-infected children and adolescents (8–17 years) on cART, HBV-negative	73 participants	HBVac (10 µg, 3-dose schedule)	Determined HBV prevalence and vaccine response in HIV-infected children on cART	7% had active HBV, 9% had past infection. Protective anti-HBs response achieved in 71% overall; 82% in children with undetectable HIV RNA, 77% in children with CD4 ≥350/mm³. HIV RNA and CD4 count predicted vaccine response. Screening for HBV prior to cART recommended.
Irungu et al., 2013 [160]	Kenya	Cohort study	HIV-1-infected and uninfected adults	603 participants (310 HIV-infected, 293 HIV-uninfected)	HBVac	Compared immune response to HBV vaccination between HIV-1-infected and uninfected adults; assessed response of HIV-infected initial nonresponders to revaccination	Nonresponse higher in HIV-infected vs uninfected (35.8% vs 14.3%, OR 3.33). Among 102 initial nonresponders, 86.3% responded to revaccination; overall response including revaccination 94.9%. Predictors of nonresponse: lower CD4 count, male sex, lower BMI, higher HIV RNA, longer time to revaccination. Timely revaccination improved overall response.
Potsch et al., 2012 [161]	Brazil	Cohort study	HIV-infected adults	163 participants	HBVac (4-double doses of 40 µg at 0, 1, 2, 6 months)	Evaluated immunogenicity and predictors of serologic response to 4-double-dose HBV vaccination in HIV-infected adults	Protective antibody response: 83% after 3 doses, 91% after 4 doses; strong antibody response ≥100 mIU/mL: 62% after 3 dos- es, 80% after 4 doses. Undetectable HIV-1 viral load and higher CD4 counts predicted stronger response. 4-double-dose regimen increased response rates and antibody titers, suggesting prolonged protection.
Launay et al., 2011 [162]	France	Random-zed controlled trial	HIV-1-infected adults, HBV seronegative, CD4 >200 cells/µL	437 enrolled, 396 followed up	HBVac; standard IM 3-dose (20 µg), 4-double-dose IM (40 µg), 4 low-dose ID (4 µg)	Compared safety and immunogenicity of alter-native HBV vaccine regimens (4 IM double doses or 4 ID low doses) versus standard 3-dose regimen	Serological response at week 28: 65% in standard IM 3-dose, 82% in 4 IM double-dose (p<0.001), 77% in 4 ID low-dose (p=0.02). No safety issues or effect on CD4/viral load. Alternative regimens improved seroconversion compared with standard schedule.
Landrum et al., 2011* [163]	USA	Cohort study	HIV-infected adults without prior HBV infection, with HAART	1,877 participants	HBVac	Evaluated timing of HBV vaccination relative to HIV diagnosis and risk of HBV infection	Vaccination completed before HIV diagnosis showed highest positive response (80%) and reduced HBV infection risk (HR=0.38) compared with unvaccinated. Vaccination after HIV diagnosis showed lower seroconversion (41%). Completing vaccine series before HIV is optimal for HBV prevention.
Flynn et al., 2011* [164]	USA	Randomized controlled trial	HIV-infected youth aged 12–<25 years, with HAART	336 participants	HBVac (Engerix B 20 µg, Engerix B 40 µg, Twinrix 20 µg HBsAg + HAV)	Compared immunogenicity of 3 HBV vaccine regimens in HIV-infected youth	Response rates: Engerix 20 µg = 60.4%, Engerix 40 µg = 73.2% (p=0.04), Twinrix = 75.4% (P=0.02). Baseline CD4+ T-cell count and vaccine regimen were independent predictors of response. Higher-dose or combination vaccines improved seroconversion.
de Vries-Sluijs et al., 2011 [165]	Netherlands	Randomized con- trolled trial	HIV-positive adults ≥18 years, HBV seronegative, not previously vaccinated	761 participants	HBVac 10 µg IM	Compared accelerated (0-1-3 weeks) vs standard (0-4-24 weeks) HBV vaccination schedules in HIV-infected adults	Overall response: standard = 50%, accelerated = 38.7% (difference 11.3%, 95% CI [4.3, 18.3]). Accelerated schedule showed higher compliance (91.8% vs 82.7%, p ≤ .001). Non-inferior efficacy in CD4+ >500 cells/mm³.

Mehta et al., 2010 [166]	USA	Cohort study	HIV-infected and HIV-uninfected adolescents	27 participants (HIV+: 12; HIV–: 15)	HBVac	Measured HSMBC following vaccination and correlation with antibody titers	HSMBC frequencies were significantly lower in HIV-infected adolescents compared with uninfected peers. Detection of an- ti-HBs ≥10 mIU/mL was associated with HSMBC. Lower HSMBC frequencies, reduced memory B cell proliferation, and altered B cell phenotypes were observed in viremic HIV-infected individuals. High antibody titers (>1000 mIU/mL) were associated with higher HSMBC in HIV-uninfected participants.
Abzug et al., 2009* [167]	USA	Cohort study	HIV-infected children on HAART	204 participants	HBVac booster	Evaluated immunogenicity and immunologic memory after HBV booster in children previously vaccinated (≥3 doses)	At entry, 24% were seropositive. Vaccine response 8 weeks post-booster occurred in 46% (seropositivity) and 37% (≥4-fold rise in anti-HBs). Immunologic memory assessed 4–5 years later was present in 45% (seropositivity) and 29% (≥4-fold rise). Higher nadir/current CD4%, higher CD19%, and undetectable HIV load predicted better response and memory. Many HIV-infected children lacked protective anti-HBs even after HAART and booster.
Launay et al., 2008 [168]	France	Randomized controlled trial	HIV-infected adults, HAV-seronegative	95 participants	HAVac	Evaluated immunogenic- ity and safety of 2-dose vs 3-dose HAV vaccination schedules in HIV-infected adults	Seroconversion at week 28: 3-dose group 82.6%, 2-dose group 69.4% (p=0.13 ITT). Observed analysis: 88.4% vs 72.3% (p=0.06). After 1 dose: 37.9%. Anti-HAV GMTs higher in 3-dose group at weeks 28 and 72 (p=0.03 and 0.05). No serious AEs. Non-smoking predicted better response (OR 2.92, p=0.04). Three doses increased antibody titers safely.
de Vries-Sluijs et al., 2008 [169]	Netherlands	Cross-sectional study	HIV-infected adults, non-responders to standard HBV vaccination	144 participants	HBVac	Evaluated efficacy of double-dose HBV revaccination in HIV-infected patients who failed previous standard vaccination	50.7% achieved seroconversion. Female patients had better response (p=0.03). Age effect depended on HIV RNA load: detectable VL—older age reduced response (OR 0.34 per 10 years, p=0.005); undetectable VL—age effect not significant (OR 0.74, p=0.12). Double-dose revaccination effective in half of prior non-responders.
Loutan et al., 2007 [170]	Switzerland	Randomized controlled trial	HIV-positive adults	14 participants	Virosomal HAVac	Evaluated immunogenicity and safety of a virosome-formulated HAV vaccine in HIV-positive adults compared to healthy adults	Seroconversion (≥20 mIU/mL) in HIV+ patients: 63.6% at Month 1, 91.7% at Month 13. GMC increased from 25.5 to 659.2 mIU/mL after booster. Well tolerated, safe, and immunogenic; lower initial response than healthy adults (93.8%→100%) but boosted effectively.
Cornejo-Juárez et al., 2006* [171]	Mexico	Randomized controlled trial	HIV-1-infected adults	79 participants	HBVac, 10 µg vs 40 µg, IM at 0, 1, 6 months	Compared immunogen- icity of two HBV vaccine doses in HIV-1-infected adults; evaluated predictors of seroconversion	Overall seroconversion: 60.7%. No significant difference be- tween 10 µg (61.5%) and 40 µg (60%) doses. CD4 ≥200 cells/mm³ strongly associated with response (86.8% vs 36.6% for <200, OR 11.44, p=0.003). Other factors (gender, age, viral load, HAART, AIDS) not associated.

Gandhi et al., 2005 [172]	USA	Cross-sectional study	HIV-1-positive adults, HBsAg- and anti-HBs-negative; subgroup positive or negative for isolated anti-HBc	69 participants	HBVac	Examined anamnestic response to HBV vaccina- tion in patients with iso- lated anti-HBc; evaluated impact of anti-HBe and HIV/HCV coinfection	Overall anamnestic response 16%; 24% in anti-HBc-positive vs 10% in anti-HBc-negative (NS). Higher response (43%) if anti-HBc + anti-HBe positive. HIV/HCV-coinfected patients less likely to achieve high anti-HBs titers. Testing for anti-HBc alone may not reliably indicate protection.
Ristola et al., 2004 [173]	Finland	Randomized controlled trial	HIV-positive adults	20 participants	HBVac, intra-dermal, 3 doses	Compared intradermal immunization with standard immunization; assessed protective antibody responses	Protective antibody (anti-HBs ≥10 IU/L or ≥4-fold rise) achieved in 39% of participants. Intradermal route induced protective immunity at similar rates to intramuscular vaccination. Suggests intradermal HBV vaccination could improve outcomes in HIV-infected adults.
Alaei et al., 2003 [174]	Iran	Cross-sectional study	HIV-positive adults	48 participants	HBVac 20 μg, 3 doses at 0, 1, 6 months	Response to conventional HBV vaccination in HIV-infected patients	Only 14/48 (29.1%) developed protective anti-HBs titers. Higher response in females (42.5%) than males (24.9%). Responders had higher mean CD4 count (351.5 vs 283.9). Lower response associated with advanced HIV stage. Authors recommend higher or booster doses, particularly in early immunologic stages.
Wilson et al., 2001 [175]	USA	Cohort study	HIV-infected and high-risk HIV-uninfected adolescents	61 (retrospective), 43 (prospective)	HBVac, 3 doses	Comparison of HBV vaccine seroresponse in HIV-infected vs high-risk HIV-uninfected adolescents	HIV-infected adolescents had lower response than HIV-uninfected (41.1% vs 70%). In prospective cohort, response rates were similar (37.1% vs 37.5%). Elevated CD8+/CD38+/HLA-DR+ T cells were associated with poor vaccine response (6.7% vs 60%), suggesting that immune activation and ongoing HIV replication impair vaccine responsiveness.
Rey et al., 2000* [176]	France	Cohort study	HIV-infected adults, CD4 >200/μL, HBV-seronegative, on stable ART	20 participants	HBVac	Effect of increasing number of HBV vaccine doses on anti-HBs response and HIV-1 viral load	After 3 doses, response rate was 55% overall, lower in CD4 200-500/μL (33.3%) vs CD4 >500/μL (87.5%). Among 9 nonresponders, 7 responded to 3 extra doses, giving an overall response of 90%. Anti-HBs titers declined over 1 year; only 58.8% maintained protective levels. Transient HIV-1 viral load in- creases observed in some patients. Doubling vaccination improved initial response but durability was limited.

* Receiving antiretroviral therapy (ART) or with highly active antiretroviral therapy (HAART)

ART – Antiretroviral therapy; CI – Confidence interval; GMC(s) – Geometric mean concentration(s); HAART – Highly active antiretroviral therapy; HAV – Hepatitis A virus; HBV – Hepatitis B virus; HAVac – Hepatitis A vaccine; HAV/Bac – Combined Hepatitis A and B vaccine; HIV – Human immunodeficiency virus; HSMBC – Hepatitis B-specific memory B cells; IFN-γ – Interferon-gamma; ITT – Intention-to-treat; LOCF – Last observation carried forward; MSM – Men who have sex with men; OR – Odds ratio; PLWH – People living with HIV; SCR – Seroconversion rate.
